# Biomimetic hydrogel design strategies for vascular grafts and vascularized tissue constructs

**DOI:** 10.3389/fbioe.2026.1839231

**Published:** 2026-07-27

**Authors:** Sen Tong, Jiaxin Chen, Yan Li, Wei Zhao

**Affiliations:** 1 Yunnan Key Laboratory of Integrated Traditional Chinese and Western Medicine for Chronic Disease in Prevention and Treatment, Yunnan University of Chinese Medicine, Kunming, China; 2 Faculty of Life Science and Technology, Kunming University of Science and Technology, Kunming, China

**Keywords:** biomimetic materials, blood vessel prosthesis, hydrogels, physiological neovascularization, tissue engineering

## Abstract

Biomimetic design strategies offer rational approaches for reconstructing functional vascular structures within hydrogel platforms. Hydrogels provide unique advantages through tissue-like hydration, tunable architectures, and biochemical functionalization capacity. These properties enable implementation of design principles derived from native vasculature. This review proposes an integrated analytical framework extracting design principles from native vascular architecture and demonstrating their application across two conventionally separate research directions, namely, vascular graft engineering and tissue vascularization. The framework encompasses four fundamental design dimensions. These are hierarchical organization spanning from arteries to capillaries, multi-layered wall architectures enabling functional stratification, biochemical microenvironments supporting vascular morphogenesis, and mechanical compliance matching physiological demands. These principles guide engineering of vascular grafts for vessel replacement and vascularized tissue constructs requiring internal perfusion. Applications include small-diameter arterial grafts, endovascular repair materials, bone tissue engineering with coupled osteogenesis and angiogenesis, chronic wound healing, and cardiac tissue regeneration. The relative weight of each design dimension varies across application contexts. Biomimetic principles function most effectively as selective design tools rather than prescriptive templates demanding maximum anatomical fidelity. Persistent challenges include temporal misalignment between scaffold degradation and vessel maturation, unpredictable anastomotic integration with host circulation, and manufacturing scalability limitations. Emerging technologies incorporating spatially controlled fabrication and stimuli-responsive behaviors offer pathways toward functional regulation beyond passive structural mimicry. This framework provides rational guidance for developing vascularized hydrogel platforms across specific therapeutic contexts.

## Graphical Abstract

**Figure:**
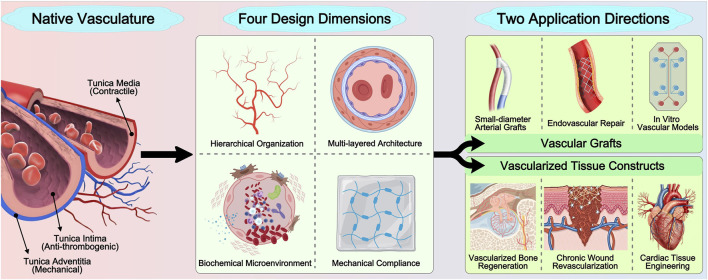

## 1 Introduction

The reconstruction of functional vascular structures within engineered biomaterials represents a fundamental challenge in regenerative medicine (Gurian et al., 2025; Liang et al., 2026). This challenge takes two distinct forms. Vascular tissue engineering seeks to fabricate blood vessel substitutes capable of restoring circulation in compromised native vessels (Zhao et al., 2024). In contrast, tissue vascularization focuses on establishing perfusable networks within engineered constructs to maintain cell viability through adequate nutrient and oxygen delivery (Wang et al., 2019). Despite serving different therapeutic purposes, both approaches share common technical requirements. They must recreate key architectural and functional characteristics of native vasculature in artificial material platforms (Gupta and Mandal, 2021). These characteristics include hierarchical network organization spanning multiple length scales and mechanical properties matched to physiological loading conditions. They also encompass biochemical microenvironments that guide vascular morphogenesis.

To address these challenges, hydrogels possess several material properties that make them particularly suitable for vascular reconstruction applications. Their high water content, typically ranging from 70% to over 95% by weight, creates physicochemical environments closely resembling native tissues. The network structure of hydrogels can be precisely controlled through adjustments in polymer concentration, crosslinking density, and fabrication parameters (Wang Y. et al., 2025). This tunability enables independent modulation of pore size, mechanical stiffness, and degradation kinetics to match specific design requirements (Yang R. et al., 2024; Liao et al., 2025). The abundant functional groups on hydrogel polymer chains facilitate covalent or non-covalent incorporation of bioactive molecules including cell adhesion peptides and growth factors. Studies have shown that natural hydrogels derived from extracellular matrix (ECM) components inherently present biological recognition sites for cell attachment and migration (Sun et al., 2023). Meanwhile, synthetic hydrogels offer reproducible properties and lower immunogenicity while allowing systematic variation of individual parameters (Wu et al., 2025). The combination of tissue-like hydration, structural adaptability, and biochemical versatility positions hydrogels as effective platforms for engineering both vascular conduits and vascularized tissue constructs.

The rational utilization of these material properties requires guidance from biological design principles. Native vascular structures have evolved structural and functional features that reflect optimization for their physiological roles. Blood vessels exhibit hierarchical organization from large arteries to capillary networks, enabling efficient distribution and exchange across multiple length scales (Llamosí et al., 2024). Vessel walls display distinct layered architectures where endothelial, smooth muscle, and adventitial components each contribute specialized functions (Jung et al., 2025). The mechanical compliance of vessels matches local hemodynamic demands, preventing stress concentrations that could trigger pathological remodeling. Biochemical microenvironments surrounding vessels present spatially organized signals that direct cell behavior during development and regeneration (Zheng et al., 2023). Understanding the functional logic underlying these native features allows extraction of biomimetic design principles. These principles can guide rational selection of materials, specification of structural parameters, and integration of biochemical cues in engineered vascular constructs. This biomimetic approach differs from empirical methods by grounding design decisions in observed structure-function relationships from natural processes.

Recent reviews have addressed hydrogel-based vascularization from various perspectives including bioprinting technologies, material selection, and applications in tissue regeneration and drug screening (Abdul Sisak et al., 2020; Barrs et al., 2020; Wang Y. et al., 2022; Nguyen et al., 2025). The present review adopts a biomimetic design framework. This framework extracts principles from native vascular architecture and demonstrates its application in both vascular grafts and vascularized tissue constructs. Although these two directions serve different therapeutic goals, they benefit from shared biomimetic strategies for recreating hierarchical organization, layered structures, biochemical signaling, and mechanical performance. The contribution of this framework lies not in proposing these four dimensions as novel concepts but in providing an integrated analytical perspective across two research directions that are conventionally treated in isolation. This perspective clarifies how the relative weight of each design dimension varies across application contexts and where shared design logic yields complementary solutions to distinct clinical challenges.

Following this conceptual foundation, this review examines native vascular architecture across four key dimensions. These dimensions include hierarchical organization, layered structure, biochemical signaling, and mechanical performance. We then demonstrate how these principles translate into practical engineering strategies. These strategies address both the construction of implantable vascular grafts and the incorporation of functional vasculature within engineered tissues. The review subsequently analyzes persistent technical challenges that limit current approaches. It also examines emerging technologies offering potential solutions and considerations for translating laboratory advances toward therapeutic applications. This framework-driven approach provides guidance for selecting materials, defining structural parameters, and integrating biochemical cues. It advances both fundamental understanding and clinical translation of vascularized hydrogel platforms.

## 2 Biomimetic design principles derived from native vasculature

### 2.1 Hierarchical organization from arteries to capillaries

Native vascular networks exhibit hierarchical organization spanning three orders of magnitude in vessel diameter. The aorta in humans measures approximately 25 mm in diameter and progressively branches into smaller arteries and arterioles. Terminal arterioles with diameters of 20–30 μm further subdivide into extensive capillary beds where individual vessels measure 5–10 μm (Hanssen et al., 2022). This stepwise reduction in caliber follows specific geometric relationships that govern branch angles and daughter vessel dimensions. The density of microvascular networks varies substantially across tissue types according to local metabolic demands. Research has demonstrated that highly metabolic organs such as myocardium and skeletal muscle contain capillary densities ranging from several hundred to over 3,000 vessels per square millimeter. Less active tissues maintain sparser networks (Noly et al., 2019; Balakrishnan et al., 2023). The spatial arrangement of these hierarchical networks ensures that no parenchymal cell resides more than 100–200 μm from the nearest capillary. This distance defines the diffusion limit for oxygen and nutrient delivery.

This hierarchical architecture reflects functional optimization for distinct physiological tasks at each scale. Large caliber vessels serve primarily as conduits for long-distance blood transport with minimal resistance. Progressive branching systematically reduces flow velocity and hydrostatic pressure. Arterial values approaching 100 mmHg decrease to capillary pressures below 30 mmHg (Richalet et al., 2024). This pressure dissipation prevents damage to delicate exchange vessels while maintaining sufficient driving force for filtration. The capillary network maximizes surface area for diffusive exchange between blood and interstitial fluid. Studies have shown that branching patterns follow principles that minimize total vascular volume while ensuring adequate perfusion of tissue volumes (Mendelson et al., 2022). The geometric parameters of vessel bifurcations distribute flow efficiently across parallel pathways, preventing regions of stagnation or excessive shear stress.

These observations yield design principles for engineering vascularized constructs in hydrogel platforms. Functional vascular networks require integration of multiple length scales ranging from macroscopic supply channels to microscopic exchange vessels. Network topology should optimize fluid distribution efficiency while minimizing total channel volume within the construct. Vessel density must match the metabolic requirements of embedded or infiltrating cells, with higher densities necessary for metabolically active tissues. The transition between scales should occur gradually through branching hierarchies rather than abrupt diameter changes that create flow disturbances. These principles apply regardless of whether the engineered construct serves as a vascular graft or as vascularized tissue. However, the specific dimensional parameters differ according to application requirements.

### 2.2 Multi-layered architecture of vessel walls

Native blood vessels possess distinct layered architectures that vary with vessel type and caliber. The innermost layer, termed tunica intima, consists of a continuous monolayer of endothelial cells resting on a basement membrane rich in type IV collagen and laminin. The middle layer or tunica media contains circumferentially arranged smooth muscle cells embedded in a matrix of elastin fibers and proteoglycans (Ding et al., 2024). The relative thickness of the media increases in arteries compared to veins, reflecting differences in pressure loading. The outermost adventitial layer comprises densely packed type I collagen fibers oriented predominantly along the vessel axis. Studies have shown that small arterioles exhibit thinner walls with fewer smooth muscle layers. In contrast, large elastic arteries contain multiple concentric lamellae of elastin alternating with smooth muscle (Ma et al., 2024). This stratified organization remains consistent across the vascular tree despite variations in absolute dimensions and layer proportions.

Each layer fulfills distinct mechanical and biological functions that collectively enable vessels to perform under physiological conditions. The endothelial monolayer prevents platelet adhesion and regulates molecular transport. Smooth muscle cells in the media modulate vessel diameter and blood flow distribution. Elastin fibers provide compliance that dampens pressure fluctuations. The collagenous adventitia limits overstretch and anchors vessels to surrounding tissues (Szklanny et al., 2021). Research indicates that for engineering vascular walls in hydrogel platforms, layered architectures should replicate this functional stratification. Material composition and cellular populations within each layer should reflect their intended functions. Examples include endothelial coverage for blood contact surfaces and mechanically robust matrices for pressure-bearing regions (Chu et al., 2025). Interfacial bonding between layers must withstand physiological stresses without delamination during cyclic loading or remodeling. The necessity of complete three-layer structures depends on application context. Vascular grafts intended for arterial replacement require robust media and adventitia to sustain hemodynamic forces. Another study found that microvascular networks within engineered tissues may function adequately with endothelial tubes supported by pericyte-like cells. Elaborate medial or adventitial components may not be necessary (Hu et al., 2024).

Design decisions should prioritize functionally relevant features over mimicry of anatomical complexity. This principle reflects a broader tension within biomimetic design between structural fidelity and functional adequacy. In contexts where manufacturing simplicity and rapid endothelialization take priority, reproducing glycocalyx function without recreating full wall stratification can achieve clinically relevant anti-thrombogenic performance (Dimitrievska et al., 2020). This outcome illustrates that functional biomimicry can satisfy therapeutic goals more efficiently than structural biomimicry under certain conditions. Where sustained hemodynamic loading is required, as in arterial graft applications, structural fidelity retains a stronger rationale because mechanical performance and biological integration both depend on approximating native wall organization. The appropriate degree of biomimetic complexity is therefore context-dependent. Introducing structural complexity beyond what a given application demands expands the parameter space requiring optimization and reduces reproducibility across fabrication batches. Biomimetic principles function most effectively as selective design tools applied according to application requirements rather than as prescriptive templates demanding maximum anatomical fidelity.

### 2.3 Biochemical microenvironment supporting vascular morphogenesis

The ECM surrounding native blood vessels presents a complex array of biochemical signals that regulate vascular cell behavior. Vascular basement membranes contain type IV collagen and laminin as major structural components. The surrounding interstitial matrix comprises type I collagen fibrils and fibronectin networks (Hallmann et al., 2020). These matrix proteins display specific amino acid sequences such as arginine-glycine-aspartate (RGD) motifs that serve as recognition sites for cellular integrin receptors (Arimori et al., 2021). Soluble growth factors exist in spatially organized gradients within this matrix environment. Research has demonstrated that vascular endothelial growth factor (VEGF) promotes endothelial cell proliferation, migration, and tube formation during angiogenic sprouting (Choi et al., 2018). Studies have shown that platelet-derived growth factor (PDGF) recruits mural cells including pericytes and smooth muscle cells to stabilize nascent vessels (Yrigoin and Davis, 2024). Additional research indicates that fibroblast growth factor (FGF) family members contribute to vessel maturation and remodeling processes (Fuchs et al., 2025). These biochemical components do not distribute uniformly. They exhibit concentration gradients and temporal variations that correspond to different stages of vascular development and maintain vessel homeostasis in mature tissues.

Matrix proteins and growth factors exert their effects through distinct but interconnected signaling mechanisms. Studies have found that ECM molecules engage integrin receptors on endothelial and mural cell surfaces. This engagement triggers intracellular cascades that regulate cytoskeletal organization, cell spreading, and directional migration (Fu et al., 2024). Growth factors bind to receptor tyrosine kinases on target cells. This binding activates downstream pathways including mitogen-activated protein kinase and phosphoinositide 3-kinase cascades that control proliferation, survival, and differentiation (Lor et al., 2025). The mode of growth factor presentation significantly influences cellular responses. Matrix-bound factors provide sustained local stimulation to cells in direct contact. Soluble gradients enable chemotactic guidance over longer distances. Research has shown that heparan sulfate proteoglycans in the ECM sequester and present growth factors in spatially controlled patterns (Mochizuki et al., 2019). The temporal coordination of these signals orchestrates the complete sequence of vascular morphogenesis. This sequence progresses from initial sprouting through lumen formation, mural cell recruitment, basement membrane deposition, and final quiescence disruption of any component in this biochemical network impairs proper vessel formation and function.

These observations establish design principles for biochemical functionalization of hydrogel-based vascular constructs. Engineered platforms should actively incorporate bioactive signals rather than relying solely on passive structural support. Cell adhesion motifs must be presented at appropriate surface densities and spatial distributions to enable cell attachment, spreading, and migration. Growth factor delivery strategies must translate these native presentation modes into engineered materials. Covalent tethering to hydrogel networks mimics matrix-bound presentation for sustained local activity. Controlled release from degradable matrices or encapsulated depots recreates soluble gradient formation. Integration of natural ECM components or decellularized matrix fragments offers complex combinatorial signals that synthetic modifications struggle to replicate. The specific biochemical design priorities differ across applications. Vascular grafts emphasize anti-thrombogenic surface properties and rapid endothelialization to prevent occlusion. Tissue vascularization applications prioritize signals that induce host vessel invasion and stabilize newly formed networks through appropriate mural cell coverage.

### 2.4 Mechanical compliance matching physiological demands

Native blood vessels exhibit nonlinear mechanical behavior characterized by progressive stiffening under increasing strain. At low strains below 30%, vessels deform relatively easily as elastin fibers within the medial layer bear the majority of tensile load. Research has shown that as strain increases beyond this threshold, collagen fibers gradually straighten and engage. This engagement causes substantial increases in stiffness that prevent overextension and potential rupture (Yao et al., 2023). This strain-hardening response protects vessels from catastrophic failure during pressure surges while maintaining adequate compliance under normal physiological conditions. The elastic modulus of blood vessels varies with anatomical location and functional requirements. Studies have demonstrated that large elastic arteries such as the aorta exhibit moduli ranging from 0.5–1.5 MPa in the physiological strain range. Muscular arteries and veins display lower stiffness values. Cerebral vessels remain particularly compliant to accommodate intracranial space constraints (Zhang et al., 2017). Blood vessels also demonstrate viscoelastic properties, dissipating mechanical energy during cyclic loading rather than behaving as purely elastic materials. This viscoelasticity arises from time-dependent rearrangement of matrix components and smooth muscle tone modulation.

These mechanical characteristics enable vessels to fulfill critical hemodynamic functions throughout the cardiovascular system. Arterial compliance allows vessel expansion during systole to accommodate stroke volume ejected by the heart. Elastic recoil during diastole continues driving blood flow forward (Gopal et al., 2025). This Windkessel mechanism smooths pulsatile flow from the heart into more continuous perfusion of peripheral tissues while reducing cardiac afterload. The strain-hardening behavior provides a safety margin against rupture during transient pressure spikes such as those occurring during physical exertion or Valsalva maneuvers (Ipar et al., 2025). Viscoelastic damping prevents resonant oscillations within the arterial tree. It also reduces mechanical fatigue of vessel walls subjected to tens of millions of loading cycles annually (Zhang et al., 2007). Mechanical matching between adjacent vessel segments ensures smooth stress transmission across junctions. Research has found that compliance mismatch at anastomotic sites generates stress concentrations. These concentrations can trigger pathological remodeling processes including intimal hyperplasia and accelerated atherosclerosis (Ozawa et al., 2020). These mechanical incompatibilities represent a major cause of synthetic graft failure in small diameter arterial applications.

These mechanical considerations establish design principles for engineering vascular constructs in hydrogel platforms. Material stiffness and compliance must match the mechanical environment where constructs will function rather than simply maximizing strength (Qu et al., 2025). Compliance mismatch between grafts and native vessels can induce maladaptive remodeling that leads to construct failure through stenosis or thrombosis. Hydrogel formulations should target nonlinear mechanical responses that mimic native tissue behavior under physiological loading conditions. Studies have shown that dual network architectures combining rigid and flexible polymer components can provide both strength and energy dissipation capacity (Wang Y. et al., 2023). Fiber reinforcement strategies enable anisotropic mechanical properties and strain-hardening responses similar to collagen engagement in natural vessels. The specific mechanical requirements vary substantially across applications. Arterial grafts must sustain pulsatile pressures exceeding 100 mmHg while maintaining compliance comparable to native arteries. Research indicates that microvascular networks within engineered tissues require sufficient mechanical integrity to resist collapse during tissue handling and remodeling. They must also remain compliant enough to deform with surrounding matrix during contraction or external compression (Yue et al., 2019). The mechanical, biochemical, and structural features described above converge on four interdependent design dimensions. These are hierarchical organization, multi-layered architecture, biochemical microenvironment, and mechanical compliance. Each dimension reflects a distinct functional requirement derived from native vascular biology. Together they constitute a unified framework capturing the essential design logic of natural vessel architecture. Table 1 summarizes the translation pathway from native vascular features to engineering design parameters across these four dimensions.

**TABLE 1:** Final biomimetic design framework translating native vascular architecture into hydrogel engineering strategies.

Design dimension	Native vascular features	Physiological functions	Engineering translation	Representative approaches	Application-specific design priority	References
Hierarchical organization	Diameter range: 25 mm to 5–10 μm; branching networks; variable capillary density	Long-distance transport, pressure dissipation, maximized exchange surface	Multi-scale networks from macro channels to micro vessels; density matched to metabolic demands	3D bioprinting, microfluidic patterning, sacrificial templating	High: bone, cardiac. Moderate: arterial grafts. Low: endovascular coatings	Hanssen et al. (2022), Mendelson et al. (2022)
Multi-layered architecture	Three distinct layers: endothelial intima, smooth muscle media, collagenous adventitia	Non-thrombogenic surface, active diameter control, mechanical support	Layered structures with composition optimized per functional requirement	Coaxial bioprinting, layer-by-layer assembly, microfluidic spinning	High: arterial grafts. Moderate: bone constructs. Low: wound dressings, endovascular coatings	Szklanny et al. (2021), Ding et al. (2024)
Biochemical microenvironment	ECM proteins with adhesion motifs; spatially organized growth factor gradients (VEGF, PDGF, FGF)	Cell adhesion, proliferation control, chemotactic guidance	Covalent tethering for sustained activity; controlled release for gradient formation	Peptide grafting, growth factor tethering/encapsulation, ECM fragment integration	High: wound healing, bone regeneration. Moderate: cardiac constructs. Low: endovascular coatings	Hallmann et al. (2020), Mochizuki et al. (2019)
Mechanical compliance	Nonlinear stress-strain behavior; modulus 0.5–1.5 MPa; viscoelastic properties	Windkessel function, rupture prevention, mechanical compatibility	Match physiological stiffness; achieve nonlinear response and strain-hardening	Dual network hydrogels, fiber reinforcement, tunable crosslinking	High: arterial grafts, cardiac constructs. Moderate: bone scaffolds. Low: wound dressings	Yao et al. (2023), Zhang et al. (2017)

3D: three-dimensional; BMP: bone morphogenetic protein; ECM: extracellular matrix; FGF: fibroblast growth factor; PDGF: platelet-derived growth factor; RGD: arginine-glycine-aspartate; VEGF: vascular endothelial growth factor.

## 3 Applications in vascular grafts and vascularized tissue constructs

Design strategies derived from native vascular architecture provide a rational basis for engineering functional blood vessel constructs in hydrogel platforms. Translating these principles into practice requires strategic integration of material formulations, fabrication technologies, and biochemical functionalization approaches. Vascular tissue engineering develops hydrogel-based grafts as implantable products that directly replace compromised blood vessels and restore circulatory function. In parallel, tissue vascularization incorporates perfusable networks within engineered constructs to sustain metabolically active cells and enable integration with host vasculature. Although these directions target different clinical needs, both rely on common design elements. These include recreating hierarchical organization, layered architectures, appropriate mechanical compliance, and instructive biochemical microenvironments derived from natural vessel design. We first explore strategies for engineering functional vascular grafts, then examine approaches to establish vascularized tissue constructs. Table 2 consolidates physiological benchmarks and engineering target ranges for key parameters across the five application scenarios discussed in this review, providing a quantitative reference against which the translational readiness of current platforms can be assessed.

**TABLE 2:** Final summary of physiological benchmarks and engineering targets across application scenarios.

Application scenario	Physiological benchmark	Engineering target	Degradation window	Vascular target and key gap	References
Small-diameter arterial grafts	Modulus 0.5–1.5 MPa; pressure above 100 mmHg	Modulus 0.5–2.0 MPa; burst pressure above 120 mmHg	Beyond 12 weeks	Three-layer wall; lumen below 6 mm; no platform satisfies all three requirements beyond 6 months	Li S. et al. (2023), Wang B. et al. (2024)
Endovascular repair materials	Near-zero friction; intima-level surface compliance	Friction approaching 0.01; anticoagulant surface activity	Permanent (coatings); 2–4 weeks (embolic agents)	Lumens as small as 0.5 mm; long-term pulsatile stability incompletely validated	Zhang M. et al. (2021), Hu et al. (2026)
Bone tissue engineering	Hierarchical vascular network; diffusion limit 200 μm	Staged VEGF and BMP delivery; macro- to micro-scale channels	8–12 weeks (vascularization); 12–24 weeks (mineralization)	Capillary spacing below 200 μm; temporal staging and clinical-scale connectivity unresolved	Hann et al. (2021), Wu et al. (2024)
Chronic wound healing	Stable capillaries within 200 μm of regenerating cells	Protease-resistant delivery; antimicrobial and angiogenic functions	2–4 weeks	Validation under pathological conditions and impaired endothelial cells absent	Shao et al. (2023), Zeng et al. (2023)
Cardiac tissue engineering	Capillary density above 3,000 vessels/mm^2^; cyclic strain above 10%	Conductive matrix; network beyond 200 μm diffusion limit	Beyond 4 weeks	Densities one order of magnitude below benchmark; integrated multi-parameter validation absent	Ng et al. (2018), Lei et al. (2019)

BMP: bone morphogenetic protein; VEGF: vascular endothelial growth factor.

### 3.1 Engineering functional vascular grafts for blood vessel replacement

#### 3.1.1 Small-diameter arterial grafts for coronary and peripheral bypass

Small-diameter arterial grafts with internal diameters below 6 mm represent the most challenging application in vascular tissue engineering (Lang et al., 2024). The reduced lumen dimensions result in lower wall shear rates and increased surface area to volume ratios. These conditions favor thrombus formation through enhanced platelet-surface interactions and reduced washout of activated coagulation factors (Ben-Shaul et al., 2019). Studies have shown that compliance mismatch between synthetic grafts and native arteries generates stress concentrations at anastomotic sites where the two materials join (Jin et al., 2023; Tang et al., 2025). These mechanical discontinuities trigger phenotypic switching of smooth muscle cells from contractile to proliferative states. This switching leads to intimal hyperplasia that progressively narrows the vessel lumen. Research has demonstrated that current synthetic materials including expanded polytetrafluoroethylene (ePTFE) and polyethylene terephthalate (PET) demonstrate acceptable performance in large diameter applications. However, synthetic grafts in small-diameter applications exhibit 5-year patency rates below 50% in peripheral bypass procedures. This contrasts sharply with autologous saphenous vein grafts, which maintain 5-year patency rates exceeding 70% in comparable settings (Li S. et al., 2023). This performance gap defines the translational failure that biomimetic hydrogel strategies must ultimately overcome. Long-term evaluation studies have emphasized that graft performance must be assessed over extended periods exceeding 6 months. This extended timeline captures remodeling processes and failure modes that emerge only during chronic implantation (Wang B. et al., 2024). The small-diameter challenge demands simultaneous achievement of anti-thrombogenicity, mechanical compatibility, and biological integration. No existing synthetic material adequately provides this combination.

**FIGURE 1:**
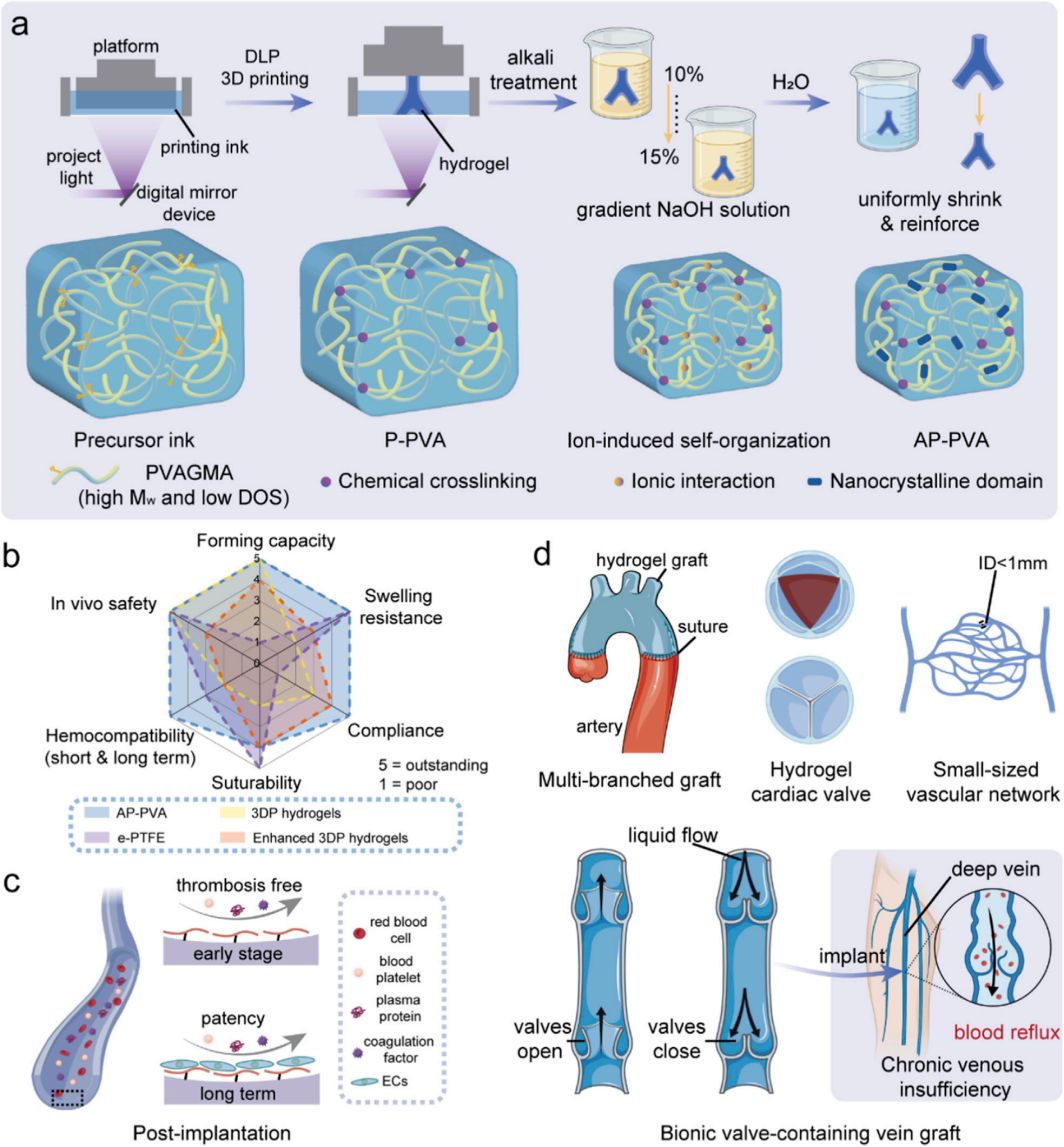
Digital light processing 3D printing produces polyvinyl alcohol hydrogel vascular constructs with integrated mechanical and biological functionality. **(a)** Fabrication process showing printing followed by nanocrystalline engineering. **(b)** Performance comparison across key parameters. **(c)** Surface-modified constructs exhibit low thrombogenicity with endothelial coverage. **(d)** Platform enables complex geometries including multi-branched grafts and valve-containing constructs. 3DP: 3D printed; AP-PVA: alkali-treated nanocrystalline polyvinyl alcohol; DLP: digital light processing; DOS: degree of substitution; ECs: endothelial cells; e-PTFE: expanded polytetrafluoroethylene; ID: inner diameter; Mw: molecular weight; P-PVA: precursor polyvinyl alcohol; PVAGMA: polyvinyl alcohol grafted with glycidyl methacrylate. Adapted from Ye et al., 2024.

Biomimetic multi-layered architectures address these requirements by recreating the functional stratification of native arterial walls. Recent studies have shown that coaxial bioprinting technologies enable fabrication of concentric tubular structures. These structures contain distinct material compositions in each layer during a single continuous process (Xu H. et al., 2022). Research has demonstrated that digital light processing three-dimensional (3D) printing of polyvinyl alcohol hydrogels produces vascular constructs. These constructs exhibit exceptional mechanical properties and biological functionality (Ye et al., 2024) (Figure 1). Nanocrystalline domain engineering through alkali treatment enhances suture tolerance and swelling resistance. The platform fabricates complex geometries including multi-branched grafts and valve-containing venous constructs. Surface modification provides low thrombogenicity while supporting endothelial layer formation for long-term patency. The outermost adventitial layer typically employs mechanically robust hydrogel formulations containing high concentrations of methacrylated gelatin or collagen. These formulations provide tensile strength and suture retention properties. The medial layer incorporates smooth muscle cells or their progenitors within moderately crosslinked hydrogels that permit cell-mediated contraction and ECM deposition. The innermost intimal layer presents endothelial cells on a compliant hydrogel surface enriched with adhesion peptides and anti-thrombogenic modifications (Cui et al., 2020). Studies have found that microfluidic spinning techniques offer an alternative approach. Coaxial flow streams of different hydrogel precursors merge within microchannel devices to form continuous hollow fibers with precisely controlled wall thickness and layer dimensions. Silk-based gel-spinning investigations have demonstrated that solution viscosity control allows tuning of scaffold porosity to balance mechanical stability with cellular colonization rates. However, excessively porous formulations may compromise long-term patency through premature mechanical failure (Rodriguez et al., 2020). Beyond these advanced fabrication approaches, numerous investigations have employed complementary strategies. These include surface coating modifications, fiber-reinforced composite structures, and decellularized matrix enhancements to achieve the multifunctional requirements of small-diameter vascular grafts. Representative platforms organized by technical strategy are summarized in Table 3. This compilation illustrates the diversity of material combinations and functionalization methods applied to address thrombosis prevention, mechanical stability, and endothelialization enhancement.

**TABLE 3:** Final summary of biomimetic hydrogel-based small-diameter vascular grafts organized by technical strategy.

Strategy category	Hydrogel composition	Reinforcement/structure	Bioactive functionalization	Validation stage	Key features	References
Surface coating approaches	Methacrylated alginate-heparin cryogel	PET fabric substrate	Heparin, RGD peptides, AAV-GFP delivery	*In vitro*	Gene therapy delivery, antithrombotic	Huynh et al. (2019)
​	HA hydrogel	Decellularized TEVG	Glycocalyx-mimetic surface	*In vivo*	Reduced platelet adhesion, macrophage inhibition	Dimitrievska et al. (2020)
​	Temperature-sensitive aliphatic polyester hydrogel	Decellularized vascular graft	VEGF conjugation	*In vivo*	Accelerated endothelialization	Iijima et al. (2018)
Bilayer composite grafts	NFC/PVA-heparin nanoparticles inner layer	PCL electrospun outer sheath	Heparin-loaded nanoparticles	*In vitro*	Anticoagulant, controlled swelling	Zhang C. et al. (2021)
​	Chitosan/silk/gelatin freeze-dried hydrogel outer	PCL/PU/gelatin electrospun inner	Heparin via emulsion	*In vitro*	Enhanced mechanical and biological properties	Almasi-Jaf et al. (2023)
​	PVA-CS-GEL hydrogel inner layer	PCL-GEL and PCL-PLA electrospun outer	Physical and chemical crosslinking	*In vitro*	Superior endothelialization	Chen et al. (2025)
Fiber-reinforced hydrogels	Ca alginate/PAAm	Braided fiber strut	—	*In vitro*	Controlled swelling, high compliance	Guan et al. (2019)
​	PVA/PAAm	Braided fiber stent	—	*In vitro*	Enhanced mechanical stability, low hemolysis	Xing et al. (2020)
​	P (AAm-co-NaAMPS)-xanthan-alginate	Double-expanded PTFE	—	*In vitro*	Improved compliance, endothelialization	Li Z. et al. (2025)
​	Poly (AAm-co-NaAMPS)-xanthan	ePTFE substrate	—	*In vitro*	Prolonged aPTT, anti-thrombotic	Li Z. et al. (2023)
Decellularized and natural matrix-based approaches	SBMA polyzwitterionic hydrogel	Interpenetrated DBIA	Hierarchical macroporous structure	*In vivo*	Complete endothelialization, M2 polarization	Wu et al. (2026)
​	Boronic acid-based PVA hydrogel	Decellularized graft perivascular coating	Rapamycin loading, tissue adhesion	*In vivo*	Prevention of anastomotic stenosis	Le et al. (2023)
​	Vascular ECM-complexed hydrogel	Heparinized PCL scaffold	Tissue-specific VECM	*In vivo*	Optimized EC and SMC regeneration	Tu et al. (2022)
​	Amniotic membrane-derived hydrogel	ADA crosslinking	REDV peptide grafting	*In vitro*	Selective EC adhesion, SMC inhibition	Peng et al. (2020)

ADA: adipic acid dihydrazide; aPTT: activated partial thromboplastin time; AAV: adeno-associated virus; CS: chondroitin sulfate; DBIA: decellularized bovine iliac artery; EC: endothelial cell; ECM: extracellular matrix; GEL: gelatin; GFP: green fluorescent protein; HA: hyaluronic acid; NaAMPS: sodium 2-acrylamido-2-methylpropane sulfonate; NFC: nanofibrillated cellulose; PAAm: polyacrylamide; PCL: polycaprolactone; PET: polyethylene terephthalate; PLA: polylactic acid; PTFE: polytetrafluoroethylene; PU: polyurethane; PVA: polyvinyl alcohol; REDV: arginine-glutamic acid-aspartic acid-valine; RGD: arginine-glycine-aspartate; SBMA: sulfobetaine methacrylate; SMC: smooth muscle cell; TEGV: tissue-engineered vascular graft; VECM: vascular extracellular matrix.

Mechanical compliance matching requires hydrogel formulations that replicate the nonlinear stress-strain behavior of native arteries while providing adequate burst strength. Research has shown that dual network hydrogels combine a rigid primary network formed through covalent crosslinking with a flexible secondary network. The secondary network assembles through physical interactions (Sun et al., 2025). This architectural strategy yields materials that exhibit low modulus at small strains for compliance. These materials resist catastrophic failure through energy dissipation in the secondary network. Fatigue resistance represents a critical but often overlooked requirement for materials subjected to millions of cardiac cycles. Recent studies have demonstrated that amphiphilic organohydrogels incorporating two-dimensional materials such as graphdiyne maintain mechanical stability over thousands of cyclic stretching cycles (Hou et al., 2022). This approach addresses the durability limitations that plague many natural hydrogel formulations. The incorporation of aligned electrospun fibers or 3D printed filaments within hydrogel matrices mimics the role of collagen fibers in native vessel walls. These reinforcing elements remain slack at low strains but progressively engage as the material stretches. This engagement produces strain-hardening responses characteristic of biological tissues (Zhou et al., 2023). Studies have found that anisotropic fiber orientation generates direction-dependent mechanical properties. Circumferential reinforcement provides hoop strength to resist radial expansion under pressure while permitting axial compliance (Gouws et al., 2024). Achieving modulus values between 0.5–2.0 MPa in the physiological strain range minimizes compliance mismatch with native coronary and peripheral arteries. This mechanical compatibility reduces stress concentrations at anastomoses that would otherwise stimulate maladaptive remodeling and graft failure.

**FIGURE 2:**
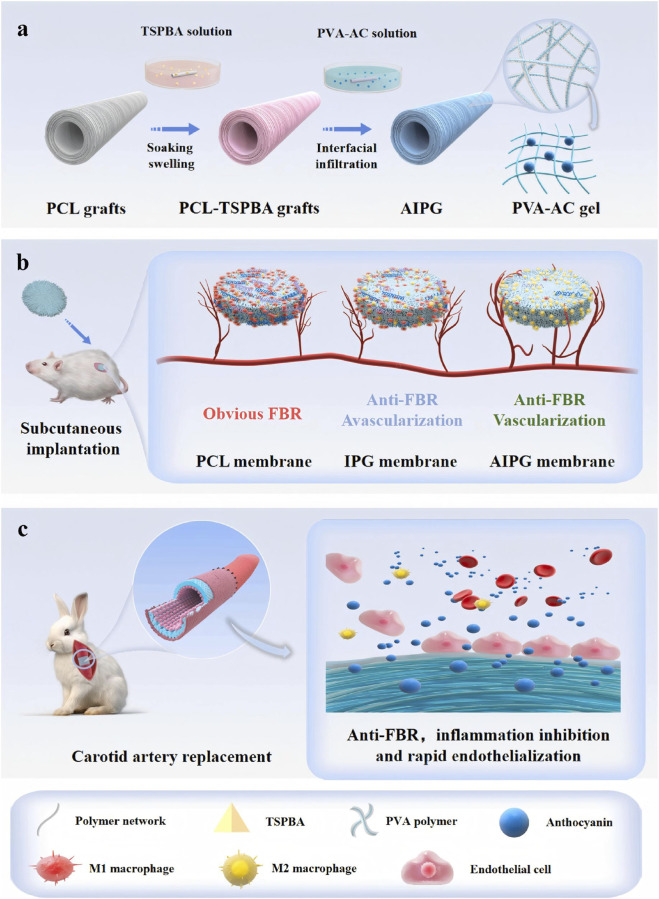
ROS-responsive hydrogel coating mitigates foreign body response and enhances vascularization in small-diameter vascular grafts. **(a)** Fabrication strategy showing interfacial infiltration of anthocyanin-loaded polyvinyl alcohol hydrogel network onto electrospun polycaprolactone substrates. **(b)** Subcutaneous implantation demonstrates superior anti-foreign body response properties and enhanced vascularization. **(c)** Rabbit carotid artery replacement model shows inflammation inhibition, endothelialization, and sustained patency. AIPG: anthocyanin-integrated polyvinyl alcohol hydrogel graft; FBR: foreign body response; IPG: interfacial polyvinyl alcohol hydrogel graft; M1/M2 macrophage: pro-inflammatory and anti-inflammatory macrophage phenotypes, respectively; ROS: reactive oxygen species; PCL: polycaprolactone; PCL-TSPBA: polycaprolactone modified with TSPBA; PVA-AC: polyvinyl alcohol loaded with anthocyanin; TSPBA: reactive oxygen species-responsive boronate ester crosslinker (N1-(4-boronobenzyl)-N3-(4-boronophenyl)-N1,N1,N3,N3-tetramethylpropane-1,3-diaminium). Adapted from Zhou M. et al., 2025.

Surface engineering strategies aim to prevent thrombosis during the critical period before complete endothelialization occurs. This strategy includes hydrophilic polymer coatings such as polyethylene glycol (PEG) or zwitterionic polymers, which create hydration layers that resist protein adsorption (Gray et al., 2018). Covalent grafting of heparin or heparin-mimetic polysaccharides provides localized anticoagulant activity at the blood-contacting interface (Zhang C. et al., 2021). Research has shown that chitosan-based hydrogels offer inherent biocompatibility and support endothelial cell adhesion under physiological shear stress conditions. These materials avoid activation of coagulation cascades, making them promising candidates for small-diameter applications (Aussel et al., 2017). Advanced functionalization strategies incorporate environmental responsiveness to address infection risks in implanted grafts. Studies have demonstrated that reactive oxygen species (ROS)-responsive approaches address foreign body reactions and oxidative stress that impair graft integration (Zhou M. et al., 2025) (Figure 2). This platform incorporates anthocyanin-loaded hydrophilic hydrogel coatings that provide stable anti-fouling interfaces and release antioxidants in response to ROS accumulation. This dual functionality mitigates inflammation and enhances vascularization, promoting rapid endothelialization and long-term patency. Peptide sequences derived from endothelial surface proteins can be tethered to hydrogel networks to present anti-thrombogenic epitopes in oriented conformations (Peng et al., 2020). However, the most effective long-term anti-thrombogenic surface remains a confluent endothelial monolayer equivalent to that lining native vessels. The integration of these surface functionalization approaches with appropriate underlying mechanical properties and layered architectures represents complete translation. This translation embodies hierarchical and mechanical design considerations in small-diameter arterial graft development.

Although the strategies in Table 3 demonstrate meaningful progress across individual performance dimensions, three gaps limit their translational readiness. Most platforms have been evaluated in animal models spanning 4–12 weeks (Fukunishi et al., 2020; Wang B. et al., 2023). This timeline falls below the 6-month minimum considered necessary to capture clinically relevant remodeling and failure modes. No hydrogel-based small-diameter graft has been compared directly against ePTFE or PET controls in an adequately powered long-term study. Second, anti-thrombogenicity, mechanical compatibility, and biological integration have largely been optimized through independent rather than integrated strategies. No reported platform has demonstrated simultaneous attainment of quantitative benchmarks across all three dimensions. Third, the fabrication approaches offering the greatest structural fidelity have not demonstrated consistent reproducibility across independent experimental settings. Among current options, acellular fiber-reinforced hydrogel composites incorporating surface functionalization represent the most tractable near-term pathway. These constructs balance mechanical performance with fabrication feasibility without requiring cell-laden processing. Achieving durable small-diameter graft function will ultimately require integrated designs validated over implantation periods that reflect clinical timelines.

#### 3.1.2 Endovascular repair materials and interventional device coatings

Endovascular interventional procedures require devices such as catheters, guidewires, and stents to navigate through tortuous vascular pathways to reach treatment sites. These devices inevitably contact vessel walls during insertion and manipulation, creating potential for mechanical injury to the endothelial lining. Studies have shown that conventional catheter materials with high surface friction coefficients or excessive rigidity can abrade the intimal layer. In extreme cases, these materials may even perforate vessel walls (Li et al., 2024). Vascular embolization represents another interventional application where materials are deliberately delivered to occlude blood flow in abnormal vessels such as arteriovenous malformations or aneurysms (Nishiguchi et al., 2025). Hydrogel-reinforced fenestrations represent a third interventional application where materials provide structural enhancement for complex multi-branched vascular repairs. In these procedures, customized endografts contain openings that align with branch vessels such as renal, superior mesenteric, or celiac arteries. Research has demonstrated that hydrogel materials applied to these fenestrations create secure seals with bridging grafts while accommodating the mechanical stresses at branch points (Azuma et al., 2022). This structural role differs from the surface modification and responsive filling functions described above, demonstrating the versatility of hydrogel platforms in endovascular interventions. These applications differ fundamentally from complete vessel replacement. The materials function temporarily during procedures or remain localized to specific anatomical sites rather than serving as permanent conduits for blood flow. The design requirements consequently emphasize surface properties and responsive behaviors rather than the comprehensive structural and mechanical specifications demanded of arterial grafts.

**FIGURE 3:**
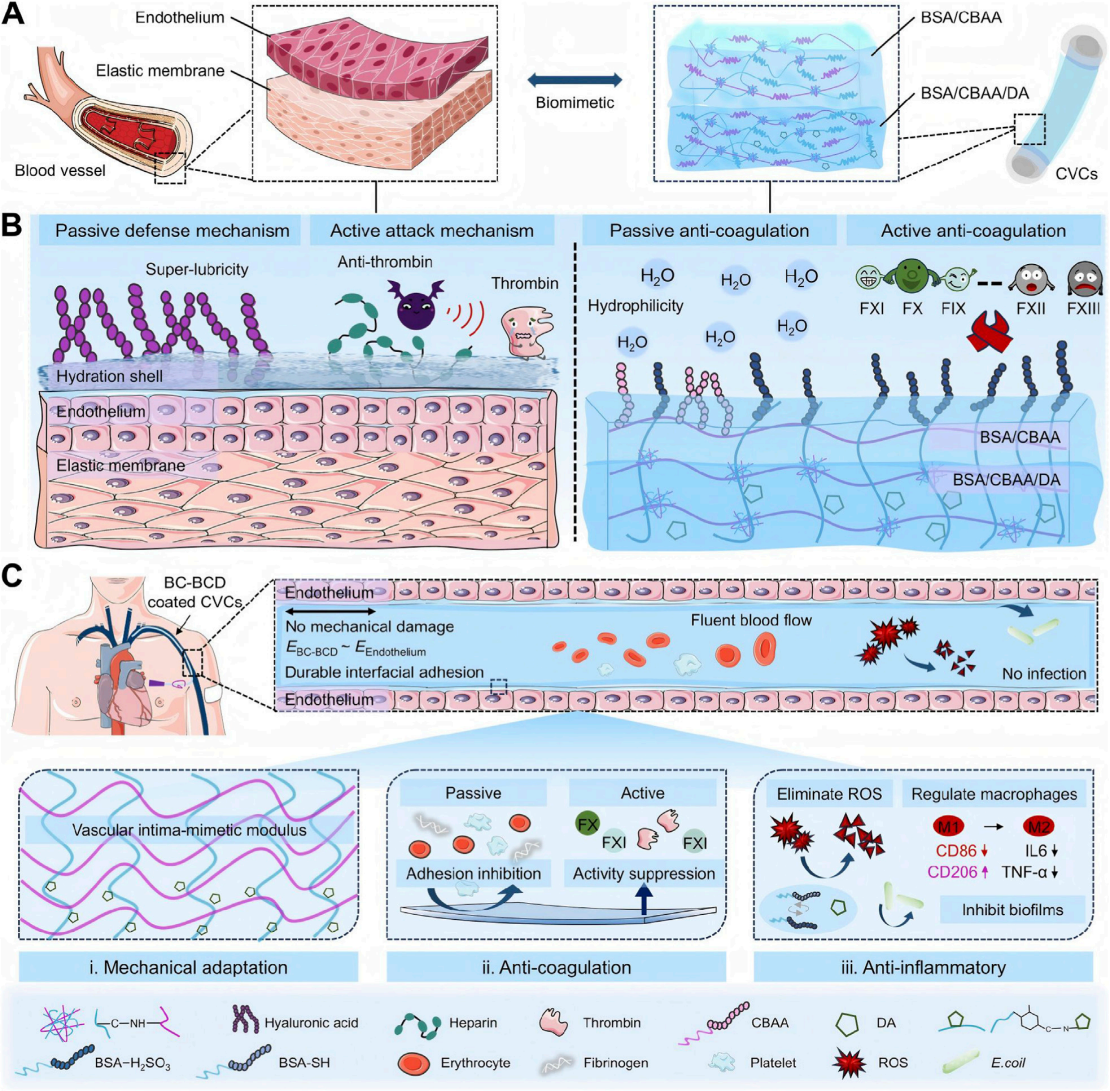
Vascular intima-biomimetic bilayer hydrogel coating provides integrated anticoagulation and inflammation regulation for central venous catheters. **(A)** Bilayer structure mimicking vascular endothelium. **(B)** Functional properties including dual-modal anticoagulation and tissue adhesion. **(C)** Mechanism for reducing catheter-related complications through coordinated thrombosis prevention and inflammatory modulation. BC-BCD: bilayer coating composed of BSA/CBAA and BSA/CBAA/DA layers; BSA: bovine serum albumin; BSA-H_2_SO_3_: sulfonated bovine serum albumin; BSA-SH: thiolated bovine serum albumin; CBAA: carboxybetaine acrylamide; CD206: cluster of differentiation 206 (M2 macrophage marker); CD86: cluster of differentiation 86 (M1 macrophage marker); CVCs: central venous catheters; DA: dopamine; *E. coli*: *Escherichia coli*; FIX, FX, FXI, FXII, FXIII: coagulation factors IX, X, XI, XII, and XIII, respectively; IL-6: interleukin-6; ROS: reactive oxygen species; TNF-α: tumor necrosis factor alpha. Adapted from Hu et al., 2026.

Hydrophilic hydrogel coatings provide lubricious surfaces that minimize frictional trauma during device manipulation within blood vessels. Studies have shown that polyvinyl alcohol hydrogels applied to catheter surfaces absorb water from the surrounding environment to form highly hydrated boundary layers (Zhang M. et al., 2021). These hydration shells reduce friction coefficients to values approaching 0.01 in aqueous media. This represents orders of magnitude improvement compared to uncoated polymer surfaces. The substantial reduction in surface friction allows devices to advance smoothly through vessels with minimal force. This decreases the risk of endothelial denudation or dissection (Gong et al., 2024). Beyond reducing mechanical trauma, the water-rich interface presented by hydrogel coatings also resists protein adsorption and platelet adhesion during limited procedural contact periods. However, indwelling catheters such as central venous catheters require more comprehensive functionalities to prevent complications during prolonged implantation periods extending weeks to months. Recent research has demonstrated that vascular intima-biomimetic bilayer hydrogel coatings address these extended requirements through integrated anticoagulation and inflammatory modulation (Hu et al., 2026) (Figure 3). The bilayer architecture provides passive anticoagulation through hydrophilic hydration shells and active inhibition via redox-responsive moieties that suppress coagulation cascades. The coating exhibits free radical scavenging capacity and macrophage phenotype remodeling to create anti-inflammatory microenvironments. The design achieves mechanical compliance matching vascular tissue while maintaining strong adhesion to catheter substrates. Clinical evaluation in arteriovenous shunt models demonstrated reduced catheter occlusion rates and suppressed inflammatory markers (Hu et al., 2026).

Injectable hydrogels serve as embolic agents for occluding abnormal vasculature in conditions including aneurysms and hypervascular tumors (Kim S. et al., 2021). These materials must remain fluid enough to pass through microcatheters during delivery and solidify rapidly upon reaching the target site to resist arterial washout. Stimuli-responsive gelation triggered by physiological temperature or ionic conditions in blood plasma provides the necessary phase transition behavior. The solidified plug must withstand pulsatile pressures exceeding 120 mmHg without fragmentation. Long-term mechanical stability requirements remain modest because thrombotic consolidation of the occluded vessel typically completes within weeks. These responsive behaviors illustrate how biomimetic design principles extend beyond structural mimicry to encompass dynamic material properties that exploit physiological conditions for functional activation.

#### 3.1.3 *In vitro* vascular models and organ-on-chip platforms for disease mechanism research

Hydrogel-based vascular constructs serve as research platforms for investigating disease mechanisms and evaluating therapeutic interventions in addition to their roles as clinical products. Studies have demonstrated that *in vitro* vascular models offer experimental advantages. These include precise control over individual variables, reproducible conditions across replicate experiments, and higher throughput compared to animal studies (Jia et al., 2019; Lan et al., 2021). These platforms also reduce the complexity inherent in whole organism experiments where multiple physiological systems interact simultaneously. However, the research value of such models depends critically on their ability to recapitulate physiologically relevant features of native vessels. The native vascular architecture described above informs the creation of experimental platforms that generate reliable data by recreating key aspects of the vascular microenvironment. Models lacking appropriate structural organization, mechanical properties, or biochemical cues may produce responses that do not translate to *in vivo* conditions. Such models provide limited insight into actual disease processes or drug efficacy.

**FIGURE 4:**
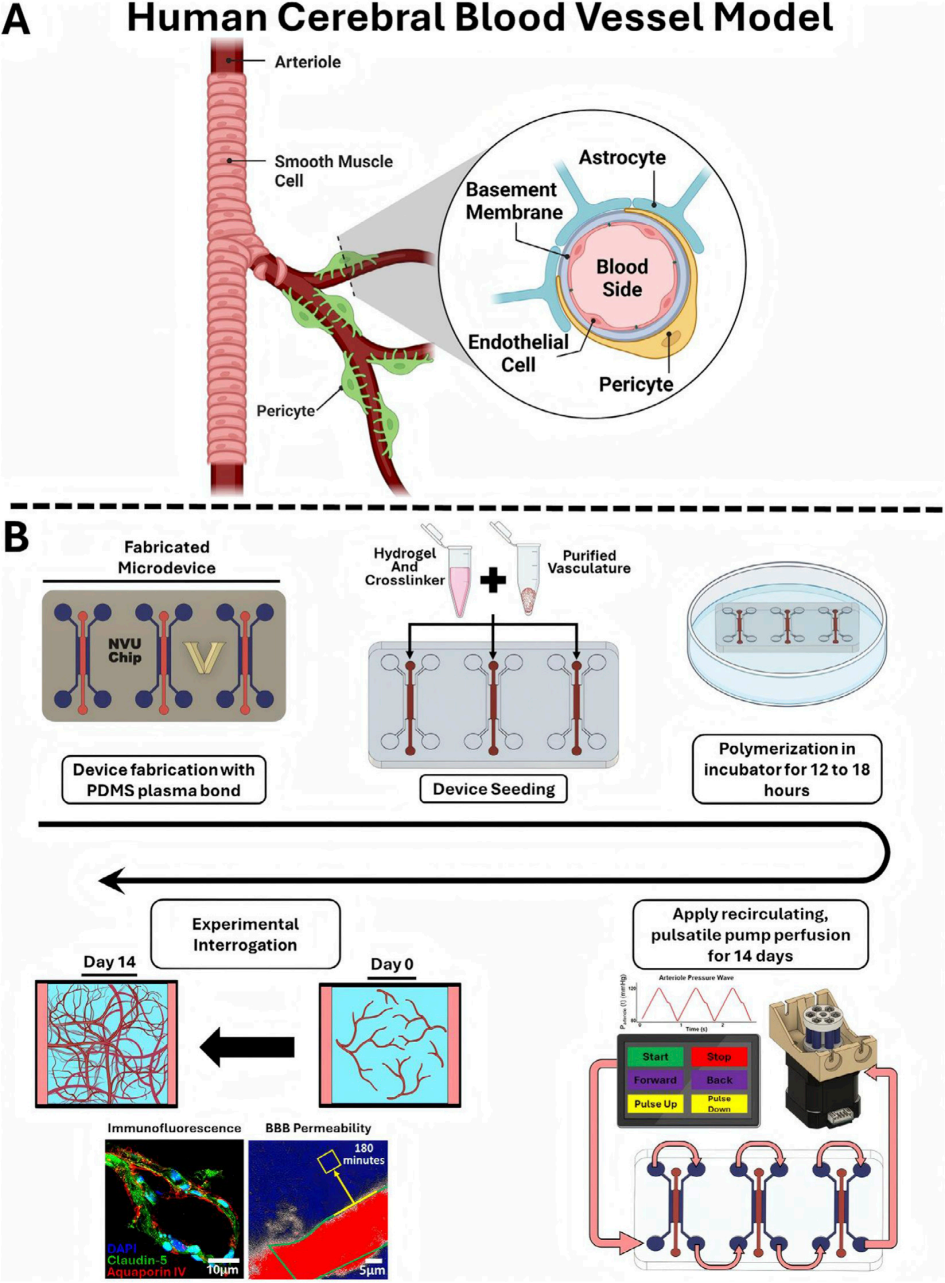
*Ex vivo* human brain vasculature-on-a-chip model preserves native neurovascular complexity and barrier function. **(A)** Neurovascular unit organization showing lumenized endothelial cells surrounded by basement membrane, pericytes, and astrocyte endfeet. **(B)** Fabrication timeline showing microdevice seeding with purified cerebral vessels in enzymatically crosslinked hydrogel followed by perfusion culture enabling vascular development within the matrix over 14 days. Aquaporin IV: a water channel protein expressed by astrocyte endfeet; BBB: blood-brain barrier; Claudin-5: a tight junction protein serving as a BBB integrity marker; NVU: neurovascular unit; PDMS: polydimethylsiloxane. Adapted from O’Grady et al., 2026.

Organ-on-chip platforms which incorporate endothelial cell-lined microfluidic channels subjected to controlled fluid flow have emerged as the primary tool for biomimetic vascular modeling in this context. This flow mimics hemodynamic conditions in native vessels (Ahmad et al., 2024). Continuous perfusion through these microchannels generates wall shear stresses ranging from 1–20 dynes/cm^2^. These values correspond to physiological flow in different vessel types. This mechanical stimulation profoundly influences endothelial cell morphology, gene expression patterns, and secretion of vasoactive factors. Studies have found that endothelial cells cultured under flow align in the direction of shear stress upregulate expression of atheroprotective genes compared to static culture conditions (Christine et al., 2016). These chips enable investigation of how flow patterns influence endothelial barrier function, inflammatory activation, and nitric oxide production. Regions of disturbed flow with low or oscillating shear stress can be engineered into chip designs to model susceptible sites for atherosclerotic lesion development. The ability to independently manipulate flow parameters while maintaining other culture conditions constant allows researchers to isolate specific biomechanical effects from biochemical variables. Advanced research has utilized purified microvessels isolated from cryopreserved human brain tissue to create authentic vascular architectures (O’Grady et al., 2026) (Figure 4). These *ex vivo* human vasculature models preserve native neurovascular unit organization including lumenized endothelial cells, basement membranes, and supporting cell populations. Following encapsulation in enzymatically crosslinked hydrogels and perfusion culture for 2 weeks, the isolated vessels demonstrate intact blood-brain barrier function. These vessels show restricted diffusion of large molecular weight tracers. This approach circumvents limitations of cell-based reconstruction by maintaining multicellular complexity and matrix microenvironments characteristic of human cerebral vasculature.

Disease-specific models recreate pathological conditions by introducing relevant molecular or cellular perturbations into these biomimetic platforms. Research has shown that vascular disease models expose endothelial cells to pathological stimuli. These stimuli include oxidized lipoproteins, inflammatory cytokines, or abnormal flow patterns to simulate disease initiation and progression (Harper et al., 2022). Studies have demonstrated that thrombosis models incorporate procoagulant factors or induce localized endothelial damage. These manipulations trigger platelet adhesion and fibrin network formation under flow conditions (Wang et al., 2014). These platforms permit real-time visualization of disease processes and quantitative assessment of therapeutic agent effects in physiologically relevant contexts. Compared to conventional static cell culture, flow-based vascular models better predict drug responses observed in clinical studies. They preserve mechanotransduction pathways and cell-cell interactions that influence treatment outcomes. The biomimetic features embedded in these research tools directly determine their translational value for understanding human vascular disease and developing effective interventions.

### 3.2 Vascularization strategies for engineered tissue constructs

#### 3.2.1 Bone tissue engineering with coupled osteogenesis and angiogenesis

Bone represents the most extensively vascularized mineralized tissue in the human body, containing vascular networks that fulfill both metabolic support and developmental regulation functions (Chen et al., 2024). Research has demonstrated that bone regeneration and vascular formation exhibit intimate spatiotemporal coupling throughout the healing cascade (Hann et al., 2021). During fracture repair, angiogenic sprouting initially establishes granulation tissue within the injury site, providing both oxygen supply and cellular recruitment pathways. Osteoprogenitor cells migrate along these newly formed vascular channels and differentiate into osteoblasts that deposit mineralized matrix. The developing bone tissue and its associated vasculature subsequently undergo coordinated remodeling to restore mechanical competence and optimize nutrient delivery (Chen et al., 2020). This coupling reflects shared molecular regulation through hypoxia-inducible factor (HIF) pathways, VEGF signaling, and bone morphogenetic protein (BMP) cascades. These pathways simultaneously promote angiogenesis and osteogenesis. Engineering strategies for bone regeneration must therefore address both processes in integrated rather than isolated approaches to recapitulate the native developmental program.

Studies have employed biomimetic hydrogel platforms combining mineral components and growth factor delivery to achieve coupled bone vascularization (Wu et al., 2024). These investigations typically utilize hyaluronic acid (HA) or gelatin methacrylate (GelMA) as base matrices. They incorporate calcium phosphate (CaP) derivatives or hydroxyapatite (HAP) to provide osteoconductivity and integrate VEGF or BMP for biochemical stimulation (Zhong et al., 2024). Individual formulations introduce additional functionalities including antioxidant activity, tissue adhesion, or antimicrobial protection tailored to specific regeneration challenges. Table 4 presents representative hydrogel platforms organized by matrix composition, illustrating how different material combinations address coupled bone vascularization challenges.

**TABLE 4:** Final summary of biomimetic hydrogel platforms for coupled bone vascularization organized by matrix composition.

Matrix category	Hydrogel matrix	Bioactive components	Special functions	Validation stage	Primary application	References
HA-based formulations	HA	—	Stem cell homing	*In vivo*	Osteoporotic bone repair	Wei et al. (2024)
​	HA/Chitosan	—	Antioxidant, angiogenic	*In vivo*	Vascularized bone regeneration	Gu et al. (2025)
​	HA	CaP	Antibacterial, neural regeneration	*In vivo*	Infected bone defect	Deng et al. (2025)
​	HA/Collagen	HAP, CaP	Biphasic osteovascular design	*In vivo*	Endochondral ossification	Kim H. D. et al. (2021)
GelMA-based formulations	GelMA/HA/Chitosan	CaP, BMP-pDNA	Sustained gene delivery	*In vivo*	Long-term osteogenesis	Huang et al. (2021)
​	GelMA/HA	Mg-modified black phosphorus	Ionic therapy	*In vivo*	Accelerated bone regeneration	Xu Y. et al. (2022)
​	GelMA/HA	VEGF, Laponite	Controlled growth factor release	*In vivo*	Vascularized bone formation	Mi et al. (2024)
Multi-component composites	Alginate/HA/Collagen	—	Nitric oxide release, self-adaptive	*In vivo*	Enhanced bone regeneration	Zhou et al. (2024)

BMP: bone morphogenetic protein; CaP: calcium phosphate; GelMA: gelatin methacryloyl; HA: hyaluronic acid; HAP: hydroxyapatite; pDNA: plasmid deoxyribonucleic acid; VEGF: vascular endothelial growth factor.

**FIGURE 5:**
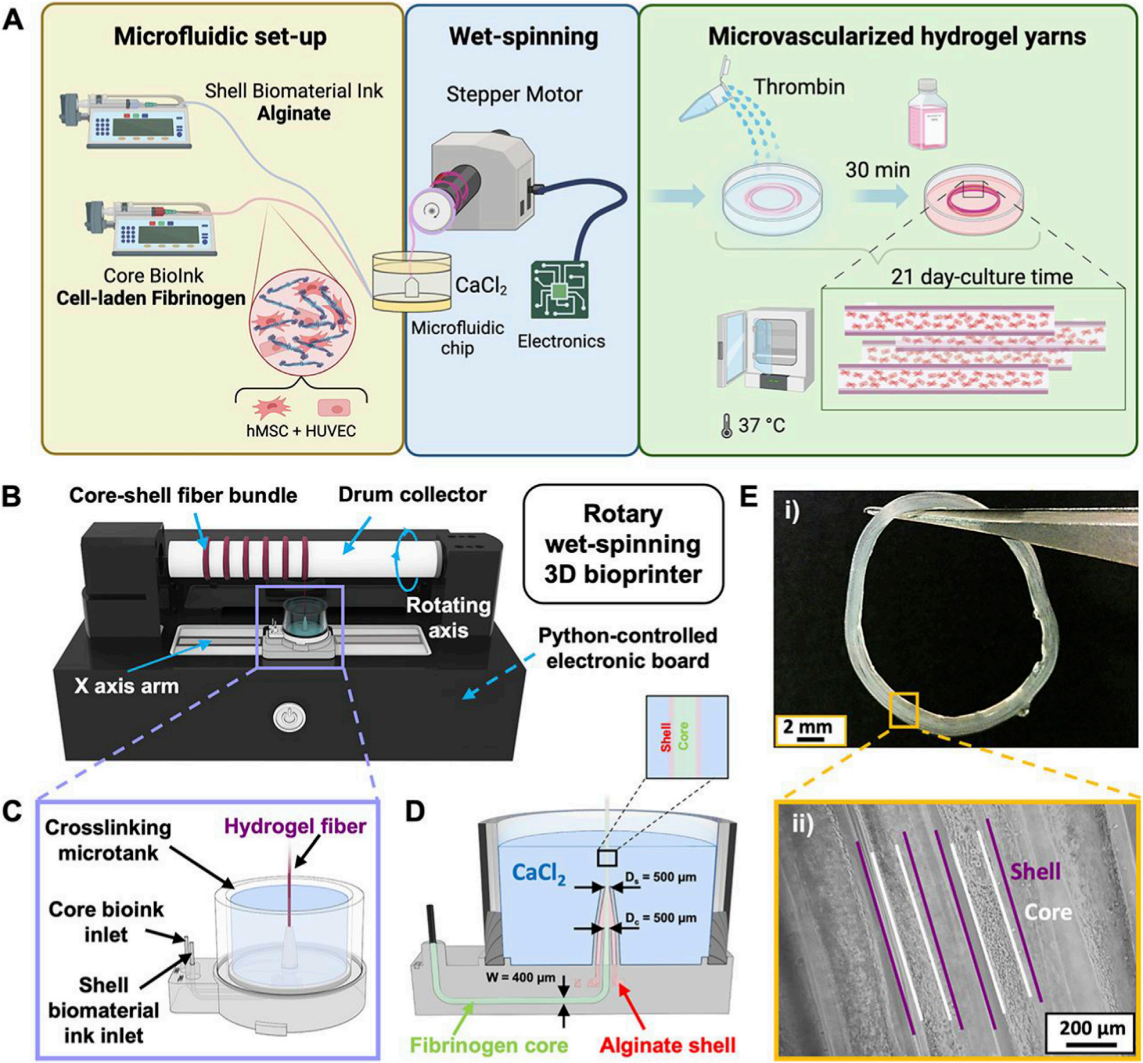
Microfluidic coaxial wet-spinning with rotary collection produces densely vascularized hydrogel fiber bundles for bone tissue engineering. **(A)** Experimental design showing automated 3D rotary wet-spinning system. **(B)** Continuous extrusion generating aligned fiber bundles. **(C)** Microfluidic printing head enabling core-shell bioink delivery. **(D)** Core-shell microchannel configuration converging at nozzle tip for fiber formation in CaCl_2_ coagulation bath. **(E)** Resulting cell-laden scaffold showing macroscopic bundle structure and highly aligned core-shell fibers with co-cultured cells. CaCl_2_: calcium chloride; hMSC: human mesenchymal stem cells; HUVEC: human umbilical vein endothelial cells. Adapted from Paradiso et al., 2024.

Advanced manufacturing technologies enable precise construction of multi-scale vascular architectures within bone scaffolds. Recent research has demonstrated that microfluidic-assisted coaxial wet-spinning combined with rotary collection fabricates densely packed hydrogel fiber bundles. These bundles contain biomimetic capillary networks (Paradiso et al., 2024) (Figure 5). The core-shell fiber architecture supports co-culture of mesenchymal stem cells and endothelial cells within a fibrin-based core surrounded by an alginate shell. This cellular organization promotes alignment along the fiber axis during extrusion and crosslinking in calcium chloride coagulation baths. Following 3 weeks of culture, oriented capillary-like networks develop within the fibers with expression of endothelial-specific markers. The rotary drum collection system creates tightly packed aligned bundles characterized by dense, extensively branched microvascular networks mimicking native bone microvasculature. Studies have shown that 3D bioprinting fabricates bone-mimetic constructs with embedded macrochannels. These channels guide formation of larger supply vessels while surrounding microporous regions accommodate capillary invasion (Anada et al., 2019). The deliberate placement of these channels optimizes nutrient diffusion distances and accelerates deep tissue vascularization compared to randomly porous structures. Research has demonstrated that four-dimensional printing extends this capability by creating personalized periosteum-like membranes. These membranes feature anisotropic microstructures that direct oriented vascular infiltration matching patient-specific defect geometries (Liu et al., 2023). Another study found that dual-mode biomanufacturing combines direct bioprinting of structural frameworks with secondary deposition of cell-laden concentrated growth factor formulations. This approach achieves both architectural control and high local bioactivity (Cui et al., 2024). Investigations have shown that programmable sacrificial bioinks undergo delayed dissolution at predetermined timepoints. This creates dynamically evolving channel networks that accommodate staged vascular development during extended culture periods (Soliman et al., 2023). These fabrication approaches integrate channels spanning millimeter to micrometer scales with spatially controlled guidance cues.

Biomimetic structural design strategies enhance vascular penetration through rational pore architecture and compositional patterning. Research has shown that phase separation void-formation technology generates interconnected macroporous networks within GelMA hydrogels. This technology simultaneously incorporates decalcified bone matrix particles for osteoinductivity and poly(lactic-co-glycolic acid) microspheres loaded with VEGF for sustained angiogenic stimulation (Wu et al., 2022). The resulting porous structure permits cellular infiltration and vascular invasion. The integrated bioactive components provide coordinated biochemical signals for coupled bone and vessel formation. Studies have demonstrated that shear-electrostatic synergistic assembly produces hydrogels with aligned biomimetic microchannels. These channels reduce diffusion distances and create preferential pathways for directional vascular ingrowth (Liu et al., 2025). Another investigation found that hamburger-like multilayer architectures mimic the nutrient periosteum surrounding native bone. These architectures present distinct compositions in each stratum to separately optimize osteogenic induction in inner layers and vascular recruitment in outer zones (Hao et al., 2024). Research has shown that hierarchical hydrogels incorporating ultralong HAP nanowires replicate the mineral organization of natural bone. The nanowire alignment generates anisotropic porosity that guides oriented vascular penetration (Geng et al., 2024). These structural innovations demonstrate how geometric and compositional organization at multiple length scales can be engineered to recapitulate the vascular-supportive architecture of native bone tissue.

**FIGURE 6:**
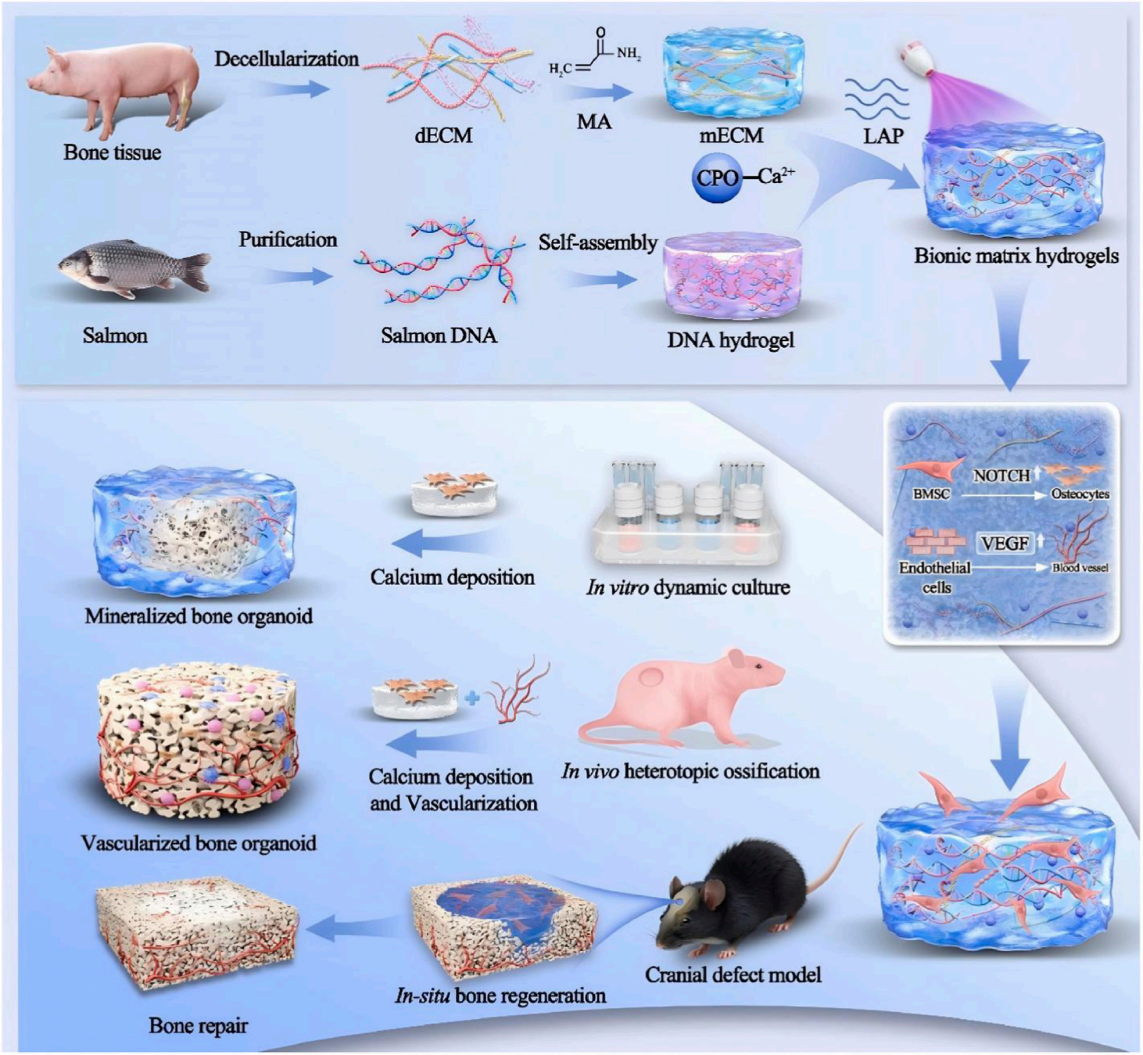
Engineered bionic matrix hydrogels enable sequential bone organoid construction with coupled vascularization and mineralization. Multifunctional components form double network structures supporting progressive organoid development that mimics bone formation. BMSC: bone marrow stem cells; CPO: calcium peroxide; dECM: decellularized extracellular matrix; LAP: lithium arylphosphinate (photoinitiator); MA: methacrylic anhydride; mECM: methacrylated extracellular matrix; NOTCH: Notch intercellular signaling pathway; VEGF: vascular endothelial growth factor. Adapted from Gai et al., 2025.

Specialized strategies integrate advanced material designs to address complex regeneration requirements. Recent studies have shown that engineered bionic matrix hydrogels incorporating multifunctional components enable sequential construction of vascularized and mineralized bone organoids (Gai et al., 2025) (Figure 6). These platforms combine decellularized extracellular matrix, deoxyribonucleic acid, and calcium phosphate oligomers through photo-crosslinking and self-assembly strategies. This creates double network architectures. The bionic matrix supports mesenchymal stromal cell differentiation and angiogenic activity. Sequential organoid development progresses through *in vitro* dynamic culture establishing prevascular networks. This is followed by *in vivo* heterotopic ossification driving mineralization. The engineered bionic matrix demonstrates efficient bone repair in cranial defect models, validating organoid-based regenerative strategies. Research has demonstrated that 3D printed ceramic scaffolds incorporating photothermal nanoparticles and chemodynamic agents combine structural support with infection control. These scaffolds also provide tissue regeneration capabilities (Lu T. et al., 2025). The photothermal component enables on-demand antibacterial action through localized hyperthermia. Chemodynamic reactions generate ROS that synergistically eliminate bacterial biofilms without impeding subsequent vascularization and osteogenesis. These multifunctional designs illustrate how biomimetic principles extend beyond structural and biochemical mimicry. They encompass integrated therapeutic functionalities that address the complex biological challenges encountered in clinical bone defects.

The platforms summarized in Table 4 collectively advance coupled bone vascularization, yet three limitations warrant explicit acknowledgment. First, native osteogenesis and angiogenesis proceed through sequential molecular events governed by HIF pathways, VEGF signaling, and BMP cascades in a defined temporal order (Hann et al., 2021; Chen et al., 2024). Most reported platforms deliver these factors simultaneously rather than in staged sequences. This approach does not faithfully recapitulate the biological program it intends to mimic. Premature BMP signaling may trigger mineralization before nascent vessels have stabilized, while sustained VEGF exposure risks disorganized angiogenic sprouting rather than structured network formation. Second, the fabrication methods demonstrating highest structural resolution perform well at sub-centimeter scales. Clinically relevant bone defects commonly occupy volumes of several cubic centimeters. Whether hierarchical vascular connectivity and cell viability can be maintained at this scale remains experimentally unresolved. Third, reported outcomes rarely include vessel density, perfusion rate, or mechanical recovery expressed against physiological benchmarks for bone tissue. This absence of standardized quantitative reporting makes cross-study comparison unreliable. Among current strategies, constructs combining staged growth factor delivery with printed macrochannel frameworks show the most coherent design logic. Validation in load-bearing large animal models represents the necessary step toward clinical relevance.

#### 3.2.2 Chronic wound healing through revascularization under pathological conditions

Chronic non-healing wounds including diabetic ulcers, burn injuries, and infected tissue defects present pathological microenvironments that actively impair vascular regeneration (Shao et al., 2023). These wounds exhibit persistent inflammation characterized by sustained neutrophil infiltration and elevated pro-inflammatory cytokine expression that disrupts normal healing progression. Bacterial colonization or biofilm formation creates additional barriers to angiogenesis through competitive consumption of oxygen and nutrients. Research has shown that tissue ischemia resulting from compromised vasculature further exacerbates the inflammatory state, creating a self-perpetuating cycle of impaired healing (Zhou E. et al., 2025). Studies have demonstrated that growth factor expression becomes dysregulated in chronic wounds. Excessive protease activity degrades endogenous VEGF and other angiogenic signals before they can effectively stimulate vessel formation (Zeng et al., 2023). Additional research has found that endothelial cell dysfunction manifests as reduced proliferative capacity, impaired migration responses, and diminished ability to organize into stable tubular structures (Lu L. et al., 2025). Newly formed vessels in these pathological contexts often exhibit abnormal permeability and insufficient pericyte coverage, leading to fragile networks that regress rather than mature. Successful vascularization strategies for chronic wounds must simultaneously address antimicrobial protection, inflammatory modulation, and angiogenic stimulation. They cannot focus on vessel formation in isolation.

Research has shown that biomimetic hydrogel platforms for wound vascularization typically employ natural polymers including collagen, chitosan, or HA as base matrices. These polymers offer inherent biocompatibility and cell-interactive properties (Huang et al., 2023; Hu et al., 2025). These formulations integrate antimicrobial agents such as metal ions or antimicrobial peptides to control infection. They also incorporate growth factors or cell-based therapies to promote vascularization and tissue regeneration (Bradley, 2020). Individual platforms address specific wound types including diabetic ulcers with metabolic dysfunction, infected wounds requiring biofilm eradication, or burn injuries demanding rapid epithelialization. Table 5 presents representative platforms organized by target application, demonstrating how platform design adapts to specific pathological wound conditions.

**TABLE 5:** Final summary of biomimetic hydrogel platforms for chronic wound revascularization organized by target application.

Matrix category	Hydrogel matrix	Bioactive components	Special functions	Validation stage	Target application	References
Diabetic wound formulations	ECM-based composite	ADSCs, polydopamine nanoparticles	Antimicrobial, stem cell delivery	*In vivo*	Diabetic foot ulcer	Li J. et al. (2025)
​	Silk nanofiber/HEC	Mg ions	Anti-inflammatory, biomechanical	*In vivo*	Diabetic wound healing	Cheng et al. (2024)
Infected wound formulations	Collagen composite	Antimicrobial peptides	Antibacterial, angiogenic	*In vivo*	Diabetic infected wound	Wang T. et al. (2024)
​	Hydrogel composite	Cu sulfide nanoparticles, deferoxamine	Antimicrobial, photothermal	*In vivo*	Diabetic infected wound	Shen et al. (2024)
General wound healing platforms	Glycopeptide hydrogel	—	Injectable, enzymatic formation	*In vitro*	Biomimetic wound healing	Qi et al. (2018)
​	Collagen-based scaffold	MSCs	Vascularized dermal graft	*In vivo*	Dermal regeneration	Li et al. (2021)
​	Alginate/Collagen	Platelet-derived factors	Natural platelet-inspired	*In vivo*	Wound healing	Jiang et al. (2022)
​	Decellularized dermal matrix	—	ECM-derived bioactivity	*In vivo*	Skin wound healing	Yu et al. (2023)
​	Glycopolypeptide hydrogel	PEGylation	Microenvironment modulation	*In vitro*	Wound healing	Teng et al. (2024)
​	Silk fibroin hydrogel	Angiogenic peptides	Injectable, biocompatible	*In vitro*	Wound vascularization	Wang et al. (2021)

ADSCs: adipose-derived stem cells; ECM: extracellular matrix; HEC: hydroxyethyl cellulose; MSCs: mesenchymal stem cells; PEG: polyethylene glycol; VEGF: vascular endothelial growth factor.

Advanced delivery technologies and physical stimulation approaches extend beyond passive growth factor release to actively modulate the wound microenvironment. Studies have demonstrated that wireless thermoelectric hydrogels generate localized electric fields that mimic the endogenous bioelectric signals present during normal wound healing. These fields promote directional endothelial cell migration and vessel sprouting (Tan et al., 2025). This approach recreates the electrical gradient that naturally develops at wound edges but becomes disrupted in chronic non-healing ulcers. Research has shown that MXene-based hydrogel microneedles enable minimally invasive delivery of triple messenger RNA (mRNA) formulations. These formulations encode pro-angiogenic and tissue remodeling factors directly into diabetic wound beds (Wang J. et al., 2025). The microneedle geometry facilitates controlled penetration depth while the MXene component provides photothermal responsiveness for on-demand payload release. Investigations have found that self-assembling peptide hydrogels present growth factors in matrix-bound configurations that protect against proteolytic degradation while enabling sustained local activity (Huang et al., 2019). The peptide sequences undergo triggered assembly into fibrillar networks upon injection. This process entraps and stabilizes growth factors through non-covalent interactions that permit gradual release as the network degrades. These sophisticated delivery strategies translate biomimetic principles of controlled spatiotemporal signaling into engineered platforms that overcome the hostile biochemical environment of chronic wounds.

Multifunctional integrated designs combine structural organization with complementary therapeutic mechanisms to address the complex pathophysiology of impaired wound healing. Studies have shown that hierarchical fiber-hydrogel dressings incorporate aligned electrospun fibers within hydrogel matrices. These structures provide both mechanical guidance for cellular organization and dual immunomodulatory functions that shift macrophage polarization from pro-inflammatory to pro-regenerative phenotypes (Wang Q. et al., 2025). The self-healing properties of these composites allow the material to maintain structural integrity despite repetitive mechanical stresses during patient movement or dressing changes. Research has demonstrated that biomimetic bilayer hydrogels loaded with stromal vascular fraction-derived exosomes combine structural design with cell-free regenerative medicine approaches. The exosomal cargo delivers coordinated pro-angiogenic and anti-fibrotic signals to promote scarless healing outcomes (Xiao et al., 2025). The bilayer configuration separates initial antimicrobial protection in the outer layer from sustained exosome release in the wound-contacting inner layer. This design optimizes the temporal sequence of therapeutic interventions.

**FIGURE 7:**
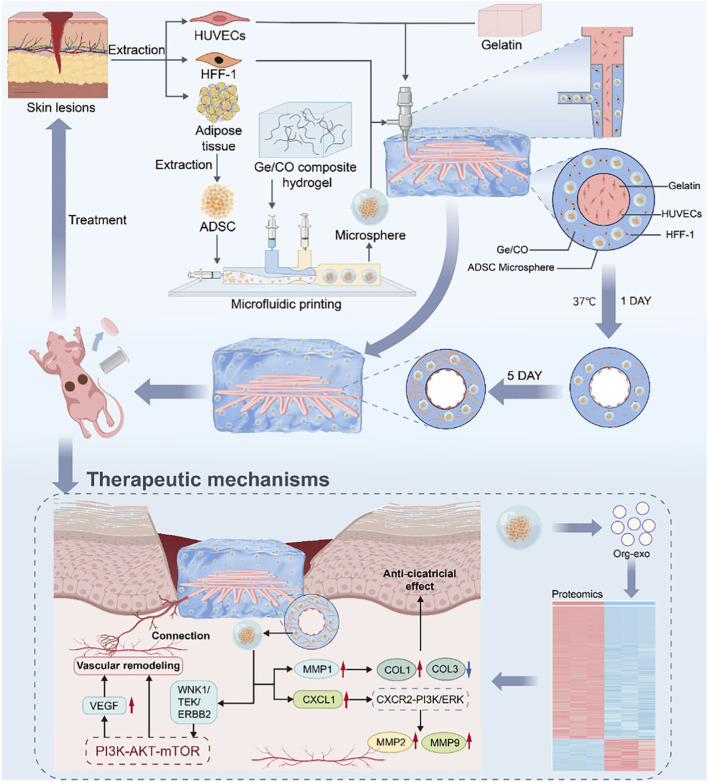
Coaxial 3D bioprinting enables one-step construction of pre-vascularized skin organoid models for wound healing. Microfluidic preparation generates adipose-derived stem cell microspheres that integrate with fibroblasts in outer hydrogel phase surrounding perfusable vascular channels formed by sacrificial inner phase. Application in full-thickness skin defects demonstrates enhanced neovascularization and wound closure. ADSC: adipose-derived stem cells; AKT: protein kinase B; COL1, COL3: collagen types I and III; CXCL1: C-X-C motif chemokine ligand 1; CXCR2: C-X-C motif chemokine receptor 2; ERBB2: erb-b2 receptor tyrosine kinase 2; ERK: extracellular signal-regulated kinase; Ge/CO: gelatin-collagen composite hydrogel; HFF-1: human foreskin fibroblast-1; HUVECs: human umbilical vein endothelial cells; MMP1, MMP2, MMP9: matrix metalloproteinases 1, 2, and 9; mTOR: mechanistic target of rapamycin; Org-exo: organoid-derived exosomes; PI3K: phosphoinositide 3-kinase; TEK: tyrosine kinase endothelial (also known as TIE2); VEGF: vascular endothelial growth factor; WNK1: with-no-lysine kinase 1. Adapted from Wang K. et al., 2025.

Specialized vascularization construction strategies harness cellular self-organization capacity or advanced manufacturing techniques to establish functional microvascular networks. Studies have shown that thermosensitive hydrogels encapsulating hemangioma stem cell-derived exosomes undergo sol-gel transition at physiological temperature. This provides a cell-free approach that exploits the potent pro-angiogenic signals naturally produced by these vascular-origin cells (Lu E. et al., 2023). Research has demonstrated that biomimetic 3D self-forming approaches utilize endothelial cell contractile forces. These forces reorganize initially homogeneous hydrogel matrices into aligned tubular structures without external templating (Zhang et al., 2020). Investigations have found that mechanotransduction-based platforms exploit reversible shear stress to regulate endothelial function. This demonstrates how physical cues can be engineered into hydrogel platforms to recapitulate the flow-responsive behavior of native vasculature (Li et al., 2026). Recent research has shown that coaxial 3D bioprinting fabricates pre-vascularized skin organoid constructs through a one-step strategy integrating vascular channels with organoid microspheres (Wang K. et al., 2025) (Figure 7). The inner phase employs temperature-responsive sacrificial material and endothelial cells forming perfusable vascular beds. The outer phase incorporates adipose-derived stem cell microspheres and fibroblasts within biomimetic hydrogel matrices. *In vivo* application in full-thickness skin defects demonstrated abundant neovessel formation, accelerated wound closure, and improved collagen remodeling through activation of pro-angiogenic signaling pathways. These diverse approaches illustrate the breadth of biomimetic strategies applicable to wound revascularization challenges.

The platforms presented in Table 5 address meaningful aspects of chronic wound pathophysiology, yet three limitations affect the interpretability of current evidence. First, most *in vitro* validation studies employ healthy endothelial cells under standard culture conditions. This does not reflect the functional impairment, elevated protease activity, and sustained hypoxia characteristic of clinical chronic wounds. Growth factor delivery systems that perform well under normal protease levels may lose biological activity rapidly within the hostile wound microenvironment. The predictive value of such *in vitro* data for clinical outcomes is therefore limited. Second, *in vivo* validation relies almost exclusively on murine full-thickness excisional models (Shao et al., 2023; Wang B. et al., 2023). Wound closure in rodents proceeds primarily through contraction rather than epithelial regeneration, diverging mechanistically from human healing. Furthermore, streptozotocin-induced diabetic models reproduce metabolic dysfunction without recapitulating the concurrent neuropathy, peripheral vascular disease, and polymicrobial biofilm infection that define refractory clinical wounds. To date, no hydrogel-based vascularization platform has demonstrated statistically significant improvements in wound closure rates or time-to-healing in adequately powered human randomized controlled trials. Available human data derive primarily from small case series and non-controlled observational studies. This evidence level is insufficient to support clinical recommendations. Third, highly integrated multifunctional platforms present an attribution challenge. When such constructs promote healing, the relative contribution of individual functional components cannot be isolated. This limits their utility for guiding rational design refinement. Among current strategies, stimuli-responsive delivery systems that exploit pathological protease activity as a triggering signal represent the most contextually coherent approach. These platforms convert a destructive feature of the wound environment into a functional driver of therapeutic release.

#### 3.2.3 Cardiac tissue engineering for myocardial regeneration

Myocardial tissue exhibits extreme metabolic activity that demands exceptionally dense vascularization to sustain contractile function (Dong et al., 2021). The human heart consumes approximately 8%–10% of total body oxygen despite representing only 0.5% of body mass. Research has shown that native myocardium contains capillary densities exceeding 3,000 vessels/mm^2^. Nearly every cardiomyocyte is positioned adjacent to at least one capillary to ensure diffusion distances remain below 10 μm (Ng et al., 2018). Cardiomyocytes demonstrate acute sensitivity to hypoxic conditions, undergoing irreversible injury within minutes of oxygen deprivation. Studies have demonstrated that engineered cardiac constructs exceeding 100–200 μm in thickness without integrated vasculature experience core necrosis. Diffusion becomes insufficient to support cell viability (Lei et al., 2019). This fundamental constraint establishes myocardial vascularization as among the most challenging applications in regenerative medicine.

**FIGURE 8:**
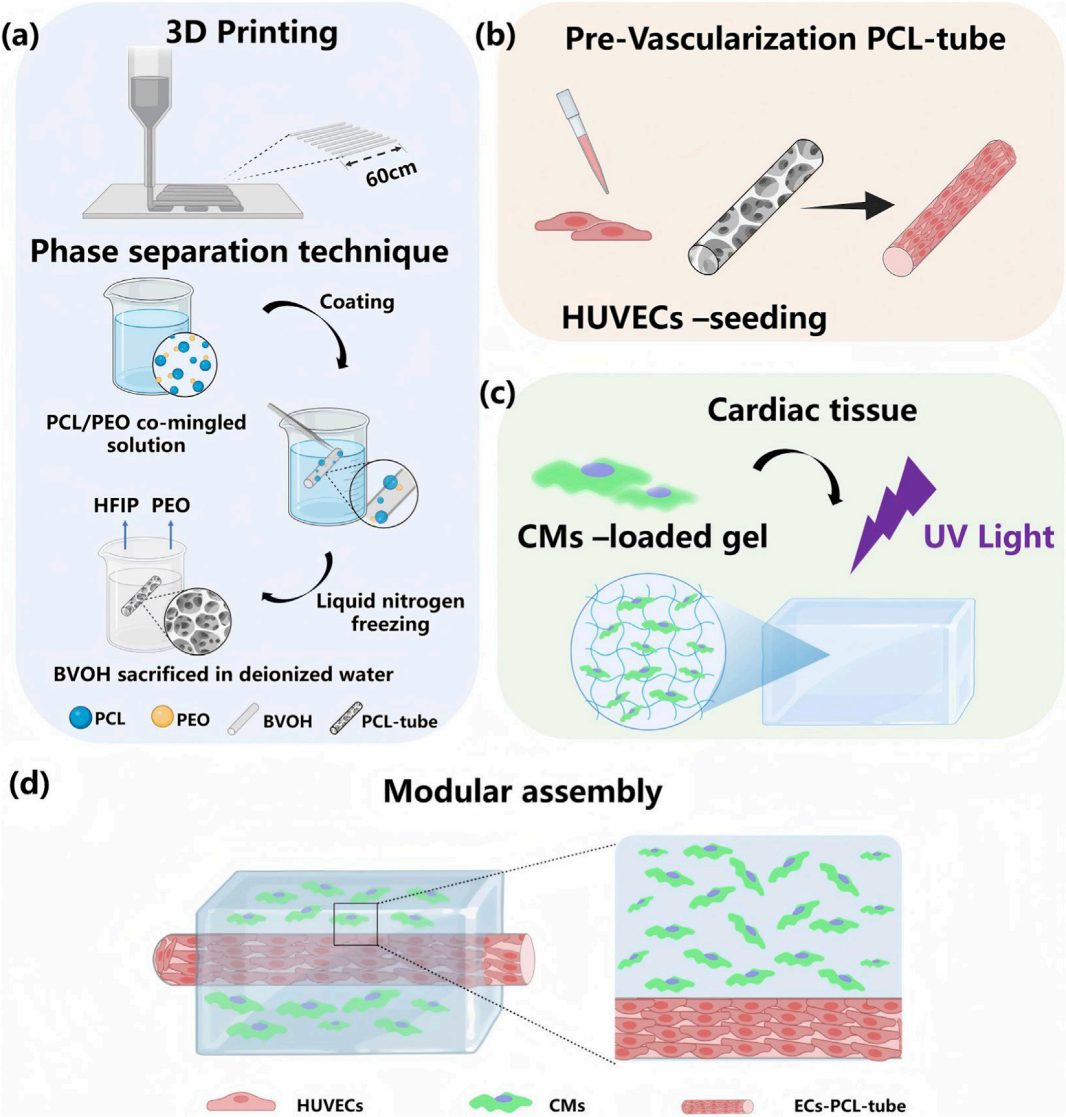
Hybrid 3D printing and phase separation enable modular assembly of biomimetic vascularized cardiac tissue. **(a)** Polycaprolactone tube fabrication providing structural support. **(b)** Endothelial cell integration forming pre-vascularized scaffolds. **(c)** Cardiomyocyte-laden fibrinogen-gelatin methacrylate hydrogel preparation. **(d)** Modular assembly strategy producing multi-scale functional cardiac tissue. BVOH: butenediol vinyl alcohol copolymer (sacrificial material); CMs: cardiomyocytes; ECs: endothelial cells; HFIP: hexafluoroisopropanol; HUVECs: human umbilical vein endothelial cells; PCL: polycaprolactone; PEO: polyethylene oxide; UV: ultraviolet light. Adapted from Pan et al., 2025.

Construction of dense microvascular networks within engineered cardiac tissues employs multiple complementary approaches. Recent research has shown that hybrid 3D printing combined with phase separation fabricates polycaprolactone tube scaffolds that provide mechanical support preventing lumen collapse (Pan et al., 2025) (Figure 8). Endothelial cells self-assemble on these structural tubes forming microvascular networks with intact barrier functions. Modular assembly integrates the endothelialized tubes with cardiomyocyte-loaded fibrinogen-gelatin methacrylate hydrogels. This creates vascularized cardiac tissue with enhanced perfusion efficiency and improved sarcomere organization. Studies have found that microfluidic patterning techniques create predefined microchannel networks mimicking native capillary bed architecture. These networks provide immediate perfusion pathways upon cellularization (Lu B. et al., 2023). These prefabricated channels can be endothelialized prior to cardiomyocyte seeding, with larger channels branching into capillary-scale vessels. Layer-by-layer assembly methods stack thin vascularized tissue sheets while maintaining vascular connectivity between layers. Research has demonstrated that coordinated delivery of multiple pro-angiogenic factors including VEGF and FGF through temporally controlled release promotes initial vessel sprouting. It also supports subsequent stabilization (Zheng et al., 2024). These approaches establish perfusion networks capable of supporting metabolically active cardiomyocyte populations.

Functional myocardial constructs require integration of vascular networks with mechanical and electrical properties necessary for synchronized contractile activity. The vascular architecture must accommodate substantial cyclic strains generated during contraction, which can exceed 10% length change in actively beating tissues. Studies have shown that hydrogel matrices incorporating fibrous reinforcement or strain-stiffening polymers provide mechanical resilience. This resilience prevents vascular collapse during repetitive contraction while maintaining sufficient compliance for tissue-level deformation (Yang P. et al., 2024). Electrically conductive hydrogels enable coordinated signal propagation throughout engineered tissue, ensuring vascular and contractile elements function in synchronized patterns. Biomimetic platforms that successfully integrate dense capillary networks with appropriate mechanical compliance and electrical conductivity represent complete translations. These translations apply multiple design principles into cardiac-specific applications.

Current platforms for engineered cardiac vascularization represent genuine technical progress, but three limitations define the distance remaining toward functional adequacy. First, native myocardium maintains capillary densities exceeding 3,000 vessels/mm^2^ (Ng et al., 2018; Noly et al., 2019). Published hydrogel-based cardiac constructs typically achieve densities one order of magnitude below this benchmark. This gap reflects a fundamental constraint of existing fabrication methods. No current approach can recreate capillary-scale network density throughout a 3D volume while simultaneously sustaining cardiomyocyte viability. This is a field-wide limitation rather than an isolated shortcoming of individual platforms. Second, functional cardiac constructs require concurrent optimization of vascular density, mechanical compliance, and electrical conductivity. These properties are almost always characterized independently across separate studies. Whether electrically driven cyclic contraction compromises vascular network integrity over physiologically relevant timescales has not been experimentally addressed. This absence of integrated multi-parameter validation limits confidence in reported construct functionality. Third, electromechanical integration between implanted constructs and host myocardium remains largely uncharacterized in existing *in vivo* studies. Among existing strategies, modular assembly approaches that mature vascular networks and cardiomyocyte compartments under separate optimized conditions before integration offer the most coherent pathway. This sequential strategy partially decouples two demanding biological processes that current single-step fabrication methods cannot simultaneously satisfy.

## 4 Current challenges and future directions

### 4.1 Persistent challenges in achieving functional vascularization

The temporal mismatch between hydrogel scaffold degradation and vascular network maturation represents a fundamental limitation in current biomimetic designs (González-Pérez et al., 2021). Newly formed vessels require several weeks to establish stable basement membranes, recruit adequate pericyte coverage, and develop mechanical resilience independent of scaffold support. Hydrogels that degrade too rapidly collapse before these maturation processes complete, causing vessel regression and tissue necrosis. Materials with excessive stability impede cellular remodeling and prevent host vessel infiltration necessary for anastomotic integration. This temporal coordination challenge extends beyond simple degradation rate tuning because different regions within a single construct experience varying cellular activities and remodeling kinetics (Fukunishi et al., 2020). The periphery contacted by host tissues may require rapid degradation to permit vascular invasion, while the core needs prolonged support until sufficient cellularity develops. Current hydrogel formulations provide uniform degradation profiles that cannot accommodate these spatially heterogeneous requirements. This results in compromised vascularization regardless of the chosen degradation rate.

Achieving functional anastomosis between engineered microvasculature and recipient circulation remains technically unresolved at clinically relevant scales (Ohayon et al., 2025). Macroscopic vascular grafts permit surgical suturing to establish blood flow continuity. In contrast, microvascular networks comprising vessels below 100 μm in diameter require spontaneous inosculation with host capillaries. This biological connection process depends on coordinated signaling between implant and host endothelial cells to guide sprouting vessels toward engineered channels (Zohar et al., 2023). The stochastic nature of angiogenic sprouting introduces unpredictable spatial patterns and temporal delays in perfusion establishment that cannot be controlled through current scaffold design strategies. Even when physical contact between vessel networks occurs, formation of patent luminal connections requires coordinated endothelial cell migration and junction remodeling. This process frequently fails to complete. Incomplete anastomosis leaves engineered vessels as isolated fluid-filled spaces without connection to systemic circulation. This negates their intended function despite correct morphological structure.

Predictive assessment of long-term vascular function in engineered constructs remains inadequate due to poor correlation between *in vitro* metrics and *in vivo* performance. Standard characterization approaches measure tubular network formation, endothelial marker expression, and perfusion of culture medium through fabricated channels (Wang X. et al., 2022). These parameters confirm structural assembly. However, they provide minimal insight into whether vessels will sustain physiological blood flow, resist thrombosis, or maintain patency over months following implantation. Animal model studies offer more physiologically relevant evaluation but exhibit species-specific healing responses and immune reactions that translate unreliably to human biology. The temporal compression inherent in rodent studies where wound healing completes in days contrasts sharply with human healing timescales spanning weeks to months (Wang B. et al., 2023). This discrepancy obscures critical failure modes that emerge only during extended remodeling periods.

### 4.2 Emerging technologies with transformative potential

Spatially controlled fabrication technologies are addressing the heterogeneity challenge by enabling region-specific material properties within single hydrogel constructs. Sacrificial bioprinting approaches deposit removable template materials that define vascular channel geometry. These templates then dissolve to leave perfusable networks embedded in structural hydrogels with independently controlled compositions (Qi et al., 2023). This two-material strategy achieves differential crosslinking densities across construct regions. Rapidly degrading peripheral zones facilitate host integration while mechanically robust cores provide sustained support during deep tissue vascularization. Microfluidic coaxial spinning produces continuous hollow fibers with graded wall compositions. Inner layers present endothelial adhesion molecules and outer layers recruit mural cells. This recreates the functional stratification of vessel walls in single-step manufacturing processes (Xie et al., 2021). These spatially resolved fabrication methods translate the hierarchical and layered design principles from native vasculature into engineered constructs. They enable regional customization that uniform hydrogel formulations cannot achieve.

Dynamic material systems that respond to biological cues offer solutions to the temporal coordination challenges limiting current static scaffolds. Enzyme-responsive hydrogels incorporate peptide crosslinkers cleaved by matrix metalloproteinases secreted during active angiogenic remodeling. This design couples degradation kinetics directly to cellular invasion rates rather than relying on predetermined hydrolysis schedules (Yang Y. et al., 2024). Integration of sensing capabilities with responsive release mechanisms creates closed-loop systems. In these systems, measured vascular density automatically modulates subsequent growth factor delivery to maintain optimal angiogenic stimulation (Mu et al., 2024). These intelligent material platforms fundamentally shift hydrogel scaffolds from passive structural templates toward active regulators. These regulators continuously adapt their properties to evolving tissue states. This transformation directly addresses the temporal mismatch and unpredictability issues that compromise static biomimetic designs. Recent work has demonstrated that combining granular hydrogel scaffolds with surgical micropuncture of recipient vessels accelerates patterned microvascular network formation within implanted biomaterials (Ataie et al., 2024). This hybrid approach, integrating material design with procedural intervention, represents a translational strategy that complements purely material-based vascularization methods.

Computational modeling approaches represent an increasingly valuable complement to experimental biomimetic strategies in vascular graft development. Computational fluid dynamics enables prediction of wall shear stress distributions and flow disturbance patterns at anastomotic sites before fabrication. Finite element analysis allows iterative optimization of graft compliance and buckling resistance across multi-dimensional design parameter spaces (Rovas et al., 2023; Szafron et al., 2024). These *in silico* methods reduce dependence on trial-and-error fabrication cycles and can identify compliance-matching configurations that minimize hemodynamic stress concentrations. Integrating computational modeling with biomimetic hydrogel design workflows offers a rational pathway for accelerating graft optimization and predicting *in vivo* performance prior to resource-intensive animal studies.

### 4.3 Pathways toward clinical translation

Transitioning biomimetic vascular hydrogel technologies from laboratory prototypes to clinical products encounters substantial manufacturing barriers that exceed purely scientific challenges (West-Livingston et al., 2023). Good Manufacturing Practice (GMP) facilities require environmental controls, equipment qualification, and quality management systems that cost millions to establish and maintain. Academic laboratories and small companies typically lack both the capital and expertise to implement these production standards independently. Hydrogel formulations containing living cells present particularly complex manufacturing requirements. Cell culture must occur under GMP conditions with documented cell banking, testing for adventitious agents, and stability validation across storage periods. Even acellular hydrogels demand rigorous characterization protocols defining critical quality attributes. These attributes include molecular weight distributions, crosslinking density, mechanical properties, endotoxin levels, and sterility across every production batch. Natural polymer extraction and processing introduce compositional variations across production lots that complicate quality control. Synthetic alternatives sacrifice biological functionality for manufacturing consistency. Scaling production from milliliter research quantities to liter clinical volumes introduces new variables affecting gelation kinetics and network structure. These variables require extensive process validation (Alonso et al., 2021). Partnering with contract development and manufacturing organizations that possess existing GMP infrastructure represents one practical mechanism for bridging this gap. Platform technology strategies that apply a single hydrogel base formulation across multiple indications can distribute foundational manufacturing development costs across broader product portfolios. The financial investment needed to establish scalable GMP manufacturing frequently exceeds the resources available before clear regulatory pathways and market opportunities justify such expenditure. This creates a circular barrier where companies cannot attract investment without manufacturing capability yet cannot build manufacturing without investment (West-Livingston et al., 2023; Alonso et al., 2021).

Regulatory pathway navigation adds a further layer of complexity that varies substantially according to product type and intended use. Topical wound dressings based on acellular hydrogel formulations typically qualify for the FDA 510(k) substantial equivalence pathway when an appropriate predicate device exists, with average review timelines of 3–12 months and relatively limited clinical data requirements. Under the European MDR 2017/745 framework, comparable products are generally classified as Class IIa or IIb medical devices, requiring clinical evaluation reports but not necessarily randomized controlled trials. Acellular implantable hydrogel grafts occupy an intermediate regulatory position, typically requiring premarket approval in the United States of America (USA), though *de novo* requests can establish novel device classifications that provide predicate foundations for subsequent similar products. Cell-containing vascularized constructs face the most demanding regulatory scrutiny. In the USA these products are classified as combination products subject to joint oversight, with development timelines from laboratory concept to regulatory approval realistically spanning fifteen to 20 years when preclinical and clinical evaluation periods are combined. Breakthrough device designation and orphan device pathways offer mechanisms for accelerated review that can meaningfully reduce both timelines and development costs for qualifying indications. Realistic clinical translation strategies should prioritize applications where biomimetic hydrogel vascularization offers decisive advantages over existing alternatives while minimizing regulatory complexity (Abdollahi et al., 2019). Wound dressings that remain external to the body face substantially lower regulatory barriers than implantable devices. These topical applications permit iterative refinement based on clinical feedback without the irreversibility of surgical procedures. Acellular formulations relying solely on biomaterial properties and incorporated growth factors avoid extensive cellular characterization and potency testing. This simplification accelerates development timelines and reduces manufacturing costs while still addressing significant therapeutic needs in chronic wound management. This phased approach builds clinical evidence and regulatory confidence incrementally rather than attempting immediate high-risk applications. Success in these accessible domains could generate the revenue and clinical data necessary to support subsequent development of more ambitious vascularized organ constructs.

## 5 Conclusion

Biomimetic design principles derived from native vascular architecture provide a unified conceptual framework for engineering functional blood vessel constructs in hydrogel platforms. This review has extracted fundamental design wisdom from natural vessels across four dimensions. These include hierarchical organization spanning multiple length scales, multi-layered wall architectures enabling functional stratification, and biochemical microenvironments orchestrating vascular morphogenesis. They also encompass mechanical compliance matching physiological loading conditions. These principles apply equally to vascular grafts intended for vessel replacement and to vascularized tissue constructs requiring internal perfusion networks. They reveal shared design challenges and complementary solutions across traditionally separate research domains. Hydrogels offer unique advantages for implementing these biomimetic strategies through their tissue-like hydration, structural tunability, and biochemical functionalization capacity. Current implementations demonstrate the value of this rational design approach in creating vascularized constructs with improved structural organization and biological integration compared to empirical material selection. However, persistent challenges continue to limit clinical translation. These include temporal misalignment between scaffold degradation and vessel maturation, unpredictable anastomotic integration with host circulation, and inadequate predictive assessment methods. Manufacturing scalability and regulatory pathway navigation present additional barriers that extend beyond purely scientific considerations.

Emerging technologies incorporating spatially controlled fabrication and stimuli-responsive behaviors represent evolution from passive structural mimicry toward active regulation of vascularization processes. Realistic translation pathways should prioritize accessible applications such as wound dressings and research platforms. These offer pragmatic routes to clinical impact while building evidence for more ambitious organ engineering applications. Progress toward these goals will require resolution of several open scientific questions that current platforms have not addressed. How hierarchical vascular connectivity can be maintained at clinically relevant tissue volumes, whether predictive *in vitro* and animal model frameworks can be established that reliably forecast long-term human performance, and how the concurrent demands of vascular density, mechanical compliance, and electrical conductivity can be reconciled within single cardiac constructs remain central challenges for the field. The fundamental value of biomimetic design lies not in slavish replication of anatomical complexity. Instead, it resides in understanding the functional logic underlying biological optimization and translating these principles into engineering parameters appropriate for specific therapeutic contexts. This understanding will continue guiding rational development of vascularized hydrogel constructs as materials science, fabrication technologies, and biological knowledge advance in concert.

## References

[B1] Abdollahi S. Boktor J. Hibino N. (2019). Bioprinting of freestanding vascular grafts and the regulatory considerations for additively manufactured vascular prostheses. Transl. Res. 211, 123–138. 10.1016/j.trsl.2019.05.005 31201778 PMC6702084

[B2] Abdul Sisak M. A. Louis F. Matsusaki M. (2020). *In vitro* fabrication and application of engineered vascular hydrogels. Polym. J. 52, 871–881. 10.1038/s41428-020-0331-z

[B3] Ahmad N. Kiriako G. Saliba J. Abla K. El-Sabban M. Mhanna R. (2024). Engineering a 3D biomimetic peptides functionalized-polyethylene glycol hydrogel model cocultured with endothelial cells and astrocytes: enhancing *in vitro* blood–brain barrier biomimicry. Mol. Pharm. 21, 4664–4672. 10.1021/acs.molpharmaceut.4c00599 39133897 PMC11372828

[B4] Almasi-Jaf A. Shamloo A. Shaygani H. Seifi S. (2023). Fabrication of heparinized bi-layered vascular graft with PCL/PU/gelatin co-electrospun and chitosan/silk fibroin/gelatin freeze-dried hydrogel for improved endothelialization and enhanced mechanical properties. Int. J. Biol. Macromol. 253, 126807. 10.1016/j.ijbiomac.2023.126807 37689302

[B5] Alonso J. M. Andrade Del Olmo J. Perez Gonzalez R. Saez-Martinez V. (2021). Injectable hydrogels: from laboratory to industrialization. Polymers 13, 650. 10.3390/polym13040650 33671648 PMC7926321

[B6] Anada T. Pan C.-C. Stahl A. M. Mori S. Fukuda J. Suzuki O. (2019). Vascularized bone-mimetic hydrogel constructs by 3D bioprinting to promote osteogenesis and angiogenesis. IJMS 20, 1096. 10.3390/ijms20051096 30836606 PMC6429349

[B7] Arimori T. Miyazaki N. Mihara E. Takizawa M. Taniguchi Y. Cabañas C. (2021). Structural mechanism of laminin recognition by integrin. Nat. Commun. 12, 4012. 10.1038/s41467-021-24184-8 34188035 PMC8241838

[B8] Ataie Z. Horchler S. Jaberi A. Koduru S. V. El‐Mallah J. C. Sun M. (2024). Accelerating patterned vascularization using granular hydrogel scaffolds and surgical micropuncture. Small 20, 2307928. 10.1002/smll.202307928 PMC11699544 37824280

[B9] Aussel A. Thébaud N. B. Bérard X. Brizzi V. Delmond S. Bareille R. (2017). Chitosan-based hydrogels for developing a small-diameter vascular graft: *in vitro* and *in vivo* evaluation. Biomed. Mat. 12, 065003. 10.1088/1748-605X/aa78d0 28604360

[B10] Azuma T. Yokoi Y. Hayakawa N. Domoto S. Niinami H. (2022). Multibranched endovascular repair using a modified endograft with hydrogel-reinforced fenestrations. Eur. J. Cardio-Thoracic Surg. 62, ezac042. 10.1093/ejcts/ezac042 35143614

[B11] Balakrishnan B. Becker C. Tenerowicz K. Puri V. (2023). Endothelial-specific expression of CIDEC protects from age-induced skeletal muscle capillary rarefaction and declined muscle performance. Physiology 38, 5795436. 10.1152/physiol.2023.38.S1.5795436

[B12] Barrs R. W. Jia J. Silver S. E. Yost M. Mei Y. (2020). Biomaterials for bioprinting microvasculature. Chem. Rev. 120, 10887–10949. 10.1021/acs.chemrev.0c00027 32867470 PMC7810139

[B13] Ben-Shaul S. Landau S. Merdler U. Levenberg S. (2019). Mature vessel networks in engineered tissue promote graft–host anastomosis and prevent graft thrombosis. Proc. Natl. Acad. Sci. U.S.A. 116, 2955–2960. 10.1073/pnas.1814238116 30718418 PMC6386696

[B14] Bradley D. (2020). Light-activated biomimetic hydrogel for infection-beating wound dressings. Mater. Today 34, 3–4. 10.1016/j.mattod.2020.02.011

[B15] Chen J. Ashames A. Buabeid M. A. Fahelelbom K. M. Ijaz M. Murtaza G. (2020). Nanocomposites drug delivery systems for the healing of bone fractures. Int. J. Pharm. 585, 119477. 10.1016/j.ijpharm.2020.119477 32473968

[B16] Chen S. Zhou X. Li T. He C. (2024). Vascularization and innervation for bone tissue engineering. Acc. Mat. Res. 5, 1121–1133. 10.1021/accountsmr.4c00165

[B17] Chen Y. Ni C. Zhou Z. Xiao J. Cao J. Pan J. (2025). Tri-layered PVA-CS-GEL hydrogel@ PCL-GEL @ PCL-PLA vascular grafts with enhanced mechanical properties and endothelialization. Compos. Commun. 60, 102638. 10.1016/j.coco.2025.102638

[B18] Cheng X. Li Y. Liang Y. Meng K. Li G. Lu Q. (2024). Mechanical biomimetic silk nano fiber-magnesium ion complex/hydroxyethylcellulose/glycerol hydrogel dressing with angiogenic capacity for accelerating scarless diabetic wound healing. J. Colloid Interface Sci. 667, 624–639. 10.1016/j.jcis.2024.03.142 38663278

[B19] Choi D. Komeda M. Heo J. Hong J. Matsusaki M. Akashi M. (2018). Vascular endothelial growth factor incorporated multilayer film induces preangiogenesis in endothelial cells. ACS Biomater. Sci. Eng. 4 (5), 1833–1842. 10.1021/acsbiomaterials.8b00100 33445338

[B20] Christine W. Xinyi J. Jianfeng L. Luke M. Christy W. Jenny P. (2016). Elucidating tumor-vasculature interactions by patterning endothelial cells into vascular-like structures in 3D biomimetic hydrogels. Front. Bioeng. Biotechnol. 4. 10.3389/conf.FBIOE.2016.01.00389

[B21] Chu S. Li L. Zhang J. You J. Li X. Zhou Y. (2025). Hierarchical interconnected porous scaffolds with regulated interfacial nanotopography exhibit antimicrobial, alleviate inflammation, neovascularization, and tissue integration for bone regeneration. Biomaterials 318, 123186. 10.1016/j.biomaterials.2025.123186 39970602

[B22] Cui H. Zhu W. Huang Y. Liu C. Yu Z.-X. Nowicki M. (2020). *In vitro* and *in vivo* evaluation of 3D bioprinted small-diameter vasculature with smooth muscle and endothelium. Biofabrication 12, 015004. 10.1088/1758-5090/ab402c 31470437 PMC6803062

[B23] Cui Z. Zhou L. Huang J. Xu L. Ding Z. Hu H. (2024). Dual-model biomanufacturing of porous biomimetic scaffolds with concentrated growth factors and embedded endothelial vascular channels for bone defect regeneration. Chem. Eng. J. 483, 148933. 10.1016/j.cej.2024.148933

[B24] Deng Y. Chen S. Tuerxun M. Xiong X. Tang J. (2025). Rapid neuralized and vascularized osteogenesis in infected bone defect using biomimetic biomineralized and antibacterial hydrogels. Front. Bioeng. Biotechnol. 13, 1611639. 10.3389/fbioe.2025.1611639 40470505 PMC12134066

[B25] Dimitrievska S. Wang J. Lin T. Weyers A. Bai H. Qin L. (2020). Glycocalyx-like hydrogel coatings for small diameter vascular grafts. Adv. Funct. Mater. 30, 1908963. 10.1002/adfm.201908963

[B26] Ding Z. Wang Y. Chen F. Hu X. Cheng W. Lu Q. (2024). Biomimetic vascular grafts with circumferentially and axially oriented microporous structures for native blood vessel regeneration. Adv. Funct. Mater. 34, 2308888. 10.1002/adfm.202308888

[B27] Dong S. Qian L. Cheng Z. Chen C. Wang K. Hu S. (2021). Lactate and myocardiac energy metabolism. Front. Physiol. 12, 715081. 10.3389/fphys.2021.715081 34483967 PMC8415870

[B28] Fu L. Bridges C. A. Kim H. N. Ding C. Bao Hou N. C. Yeow J. (2024). Cationic polysaccharides bind to the endothelial cell surface extracellular matrix involving heparan sulfate. Biomacromolecules 25, 3850–3862. 10.1021/acs.biomac.4c00477 38775104

[B29] Fuchs M. A. A. Burke E. J. Latic N. Murray S. L. Li H. Sparks M. A. (2025). Fibroblast growth factor 23 and fibroblast growth factor receptor 4 promote cardiac metabolic remodeling in chronic kidney disease. Kidney Int. 107, 852–868. 10.1016/j.kint.2025.01.024 39923962 PMC12755233

[B30] Fukunishi T. Ong C. S. Yesantharao P. Best C. A. Yi T. Zhang H. (2020). Different degradation rates of nanofiber vascular grafts in small and large animal models. J. Tissue Eng. Regen. Med. 14, 203–214. 10.1002/term.2977 31756767

[B31] Gai T. Zhang H. Hu Y. Li R. Wang J. Chen X. (2025). Sequential construction of vascularized and mineralized bone organoids using engineered ECM-DNA-CPO-based bionic matrix for efficient bone regeneration. Bioact. Mater. 49, 362–377. 10.1016/j.bioactmat.2025.02.033 40144795 PMC11937690

[B32] Geng X. Tang Y. Yuan B. Dai Y. Yu H.-P. Xiong Z.-C. (2024). Biomimetically ordered ultralong hydroxyapatite nanowires-based hierarchical hydrogel scaffold with osteoimmunomodulatory and osteogenesis abilities for augmenting bone regeneration. Chem. Eng. J. 488, 151136. 10.1016/j.cej.2024.151136

[B33] Gong Y. Guo J. Xiang Y. Liang W. Li S. Leng Y. (2024). Polydopamine-polyvinyl alcohol hydrogel coatings with enhanced mechanical and tribological performance. Prog. Org. Coatings 197, 108812. 10.1016/j.porgcoat.2024.108812

[B34] González-Pérez F. Ibáñez-Fonseca A. Alonso M. Rodríguez-Cabello J. C. (2021). Combining tunable proteolytic sequences and a VEGF-mimetic peptide for the spatiotemporal control of angiogenesis within Elastin-like recombinamer scaffolds. Acta Biomater. 130, 149–160. 10.1016/j.actbio.2021.06.005 34118450

[B35] Gopal K. Choi S. Y. Lee H. J. Ko S. W. Krishnamoorthi Kaliannagounder V. Park C. H. (2025). Smart triboelectric bilayer vascular graft with integrated drug delivery and real-time hemodynamic sensing. Nano Energy 144, 111391. 10.1016/j.nanoen.2025.111391

[B36] Gouws X. A. Mastnak A. Kreplak L. Rutenberg A. D. (2024). Anisotropic swelling due to hydration constrains anisotropic elasticity in biomaterial fibers. J. Mech. Behav. Biomed. Mater. 160, 106749. 10.1016/j.jmbbm.2024.106749 39317097

[B37] Gray W. A. Keirse K. Soga Y. Benko A. Babaev A. Yokoi Y. (2018). A polymer-coated, paclitaxel-eluting stent (Eluvia) *versus* a polymer-free, paclitaxel-coated stent (Zilver PTX) for endovascular femoropopliteal intervention (IMPERIAL): a randomised, non-inferiority trial. Lancet 392, 1541–1551. 10.1016/S0140-6736(18)32262-1 30262332

[B38] Gu G. Xu Y. He P. Li C. Chen S. Wei G. (2025). Bioinspired composite hydrogels with osteogenic, angiogenic, and antioxidant properties for enhanced bone repair. Small Struct. 6, 2400462. 10.1002/sstr.202400462

[B39] Guan G. Yu C. Xing M. Wu Y. Hu X. Wang H. (2019). Hydrogel small-diameter vascular graft reinforced with a braided fiber strut with improved mechanical properties. Polymers 11, 810. 10.3390/polym11050810 31064087 PMC6571729

[B40] Gupta P. Mandal B. B. (2021). Silk biomaterials for vascular tissue engineering applications. Acta Biomater. 134, 79–106. 10.1016/j.actbio.2021.08.004 34384912

[B41] Gurian M. Allijn I. E. Veenendaal L. Bassous N. Shin S. R. Leijten J. (2025). Self‐feeding of engineered tissues via controlled glucose release facilitates survival and vascularization of living implants. Adv. Funct. Mater. 36, e28636. 10.1002/adfm.202528636

[B42] Hallmann R. Hannocks M.-J. Song J. Zhang X. Di Russo J. Luik A.-L. (2020). The role of basement membrane laminins in vascular function. Int. J. Biochem. and Cell Biol. 127, 105823. 10.1016/j.biocel.2020.105823 32781135

[B43] Hann S. Y. Cui H. Esworthy T. Zhou X. Lee S. Plesniak M. W. (2021). Dual 3D printing for vascularized bone tissue regeneration. Acta Biomater. 123, 263–274. 10.1016/j.actbio.2021.01.012 33454383

[B44] Hanssen H. Streese L. Vilser W. (2022). Retinal vessel diameters and function in cardiovascular risk and disease. Prog. Retin. Eye Res. 91, 101095. 10.1016/j.preteyeres.2022.101095 35760749

[B45] Hao S. Zhou D. Wang F. Li G. Deng A. Ren X. (2024). Hamburger-like biomimetic nutrient periosteum with osteoimmunomodulation, angio-/osteo-genesis capacity promoted critical-size bone defect repair. Chem. Eng. J. 489, 150990. 10.1016/j.cej.2024.150990

[B46] Harper R. L. Ferrante E. A. Boehm M. (2022). Development of vascular disease models to explore disease causation and pathomechanisms of rare vascular diseases. Semin. Immunopathol. 44, 259–268. 10.1007/s00281-022-00925-9 35233690 PMC8887661

[B47] Hou J. Zhang X. Wu Y. Jie J. Wang Z. Chen G.-Q. (2022). Amphiphilic and fatigue-resistant organohydrogels for small-diameter vascular grafts. Sci. Adv. 8, eabn5360. 10.1126/sciadv.abn5360 35905180 PMC9337766

[B48] Hu Y. Li L. Li Q. Pan S. Feng G. Lan X. (2024). A biomimetic tri-phasic scaffold with spatiotemporal patterns of gastrodin to regulate hierarchical tissue-based vascular regeneration. Bioact. Mater. 38, 512–527. 10.1016/j.bioactmat.2024.05.007 38798891 PMC11126808

[B49] Hu F. He Y. Wang Q. Duan X. Liu J. Qiu H. (2025). Bionic hydrogel treatments for diabetic wounds. Apl. Mater. 13, 060603. 10.1063/5.0274646

[B50] Hu X. Mou X. Pan G. Liu Y. Song W. Xiao P. (2026). Vascular intima-biomimetic bilayer hydrogel coating of central venous catheters with dual-modal anti-coagulation and self-adaptive immunomodulation. Adv. Funct. Mater. 36, e11029. 10.1002/adfm.202511029

[B51] Huang L.-C. Wang H.-C. Chen L.-H. Ho C.-Y. Hsieh P.-H. Huang M.-Y. (2019). Bioinspired self-assembling peptide hydrogel with proteoglycan-assisted growth factor delivery for therapeutic angiogenesis. Theranostics 9, 7072–7087. 10.7150/thno.35803 31660087 PMC6815956

[B52] Huang L. Zhang Z. Guo M. Pan C. Huang Z. Jin J. (2021). Biomimetic hydrogels loaded with nanofibers mediate sustained release of pDNA and promote *in situ* bone regeneration. Macromol. Biosci. 21, 2000393. 10.1002/mabi.202000393 33625790

[B53] Huang C. Dong L. Zhao B. Huang S. Lu Y. Zhang X. (2023). Tunable sulfated Alginate-based hydrogel platform with enhanced anti-inflammatory and antioxidant capacity for promoting burn wound repair. J. Nanobiotechnol 21, 387. 10.1186/s12951-023-02144-2 PMC10594798 37875922

[B54] Huynh C. Shih T.-Y. Mammoo A. Samant A. Pathan S. Nelson D. W. (2019). Delivery of targeted gene therapies using a hybrid cryogel-coated prosthetic vascular graft. PeerJ 7, e7377. 10.7717/peerj.7377 31497383 PMC6707340

[B55] Iijima M. Aubin H. Steinbrink M. Schiffer F. Assmann A. Weisel R. D. (2018). Bioactive coating of decellularized vascular grafts with a temperature-sensitive VEGF-conjugated hydrogel accelerates autologous endothelialization *in vivo:* hydrogel-conjugated VEGF accelerates autologous endothelialization. J. Tissue Eng. Regen. Med. 12, e513–e522. 10.1002/term.2321 27689942

[B56] Ipar E. Cymberknop L. J. Elizondo M. Gerona S. Armentano R. L. (2025). Cardiac hemodynamic status: an interpretable one-look Windkessel index of arterial health. Am. J. Physiol.-Heart Circulatory Physiol. 329, H1716–H1724. 10.1152/ajpheart.00771.2025 41264378

[B57] Jia L. Han F. Yang H. Turnbull G. Wang J. Clarke J. (2019). Microfluidic fabrication of biomimetic helical hydrogel microfibers for blood-vessel-on-a-chip applications. Adv. Healthc. Mater. 8, 1900435. 10.1002/adhm.201900435 31081247

[B58] Jiang Y. Wang J. Zhang H. Chen G. Zhao Y. (2022). Bio-inspired natural platelet hydrogels for wound healing. Sci. Bull. 67, 1776–1784. 10.1016/j.scib.2022.07.032 36546063

[B59] Jin Q. Yu C. Xu L. Zhang G. Ju J. Hou R. (2023). Combined light-cured and sacrificial hydrogels for fabrication of small-diameter bionic vessels by 3D bioprinting. THC 31, 1203–1213. 10.3233/THC-220393 36872804

[B60] Jung C. Han J. W. Lee S.-J. Kim K. H. Oh J. E. Bae S. (2025). Novel directly reprogrammed smooth muscle cells promote vascular regeneration as microvascular mural cells. Circulation 151, 1076–1094. 10.1161/CIRCULATIONAHA.124.070217 39945059 PMC11996609

[B61] Kim H. D. Hong X. An Y. Park M. J. Kim D. Greene A. K. (2021). A biphasic osteovascular biomimetic scaffold for rapid and self-sustained endochondral ossification. Adv. Healthc. Mater. 10, 2100070. 10.1002/adhm.202100070 PMC8273143 33882194

[B62] Kim S. Nowicki K. W. Gross B. A. Wagner W. R. (2021). Injectable hydrogels for vascular embolization and cell delivery: the potential for advances in cerebral aneurysm treatment. Biomaterials 277, 121109. 10.1016/j.biomaterials.2021.121109 34530233

[B63] Lan D. Shang Y. Su H. Liang M. Liu Y. Li H. (2021). Facile fabrication of hollow hydrogel microfiber via 3D printing-assisted microfluidics and its application as a biomimetic blood capillary. ACS Biomater. Sci. Eng. 7, 4971–4981. 10.1021/acsbiomaterials.1c00980 34503336

[B64] Lang Z. Chen T. Zhu S. Wu X. Wu Y. Miao X. (2024). Construction of vascular grafts based on tissue-engineered scaffolds. Mater. Today Bio 29, 101336. 10.1016/j.mtbio.2024.101336 39624049 PMC11609327

[B65] Le H. T. Mahara A. Nagasaki T. Yamaoka T. (2023). Prevention of anastomotic stenosis for decellularized vascular grafts using rapamycin-loaded boronic acid-based hydrogels mimicking the perivascular tissue function. Biomater. Adv. 147, 213324. 10.1016/j.bioadv.2023.213324 36796198

[B66] Lei D. Yang Y. Liu Z. Yang B. Gong W. Chen S. (2019). 3D printing of biomimetic vasculature for tissue regeneration. Mat. Horiz. 6, 1197–1206. 10.1039/C9MH00174C

[B67] Li X. You R. Zhang Q. Yan S. Luo Z. Qu J. (2021). Engineering vascularized dermal grafts by integrating a biomimetic scaffold and Wharton’s jelly MSC-derived endothelial cells. J. Mat. Chem. B 9, 6466–6479. 10.1039/D1TB00857A 34364307

[B68] Li S. Zhao F. Tang Y. Zhang Y. Rong H. Liu L. (2023). Bioinspired, Anticoagulative,^19^ F MRI-Visualizable bilayer hydrogel tubes as high patency small-diameter vascular grafts. Small 19, 2302621. 10.1002/smll.202302621 37340585

[B69] Li Z. Giarto J. Zhang J. Kulkarni N. Mahalingam E. Klipstine W. (2023). Anti-thrombotic poly(AAm-co-NaAMPS)-xanthan hydrogel-expanded polytetrafluoroethylene (ePTFE) vascular grafts with enhanced endothelialization and hemocompatibility properties. Biomater. Adv. 154, 213625. 10.1016/j.bioadv.2023.213625 37722163 PMC10841274

[B70] Li P. Zhang X. Feng J. Yu C. Fang D. Zhang J. (2024). Preparation and performance verification of polyvinyl alcohol hydrogel biomimetic coating on the surface of vascular intervention guidewires. Colloids Surfaces A Physicochem. Eng. Aspects 701, 134949. 10.1016/j.colsurfa.2024.134949

[B71] Li J. Wang F. Ding Y. Chen G. Tan Q. Zhao Y. (2025). Biomimetic ECM hydrogels loaded with ADSCs and polydopamine nanoparticles for chronic wound healing. Chem. Eng. J. 507, 160764. 10.1016/j.cej.2025.160764

[B72] Li Z. Chen E. Parsons J. Turng L. (2025). Design, synthesis, and characterization of polymer-hydrogel composite vascular grafts using double-expanded polytetrafluoroethylene to achieve enhanced mechanical and biological properties. Polym. Eng. and Sci 65, 2893–2908. 10.1002/pen.27185

[B73] Li Z. Li S. Cui R. Jing T. Peng H. Wang R. (2026). Reversible shear stress-mediated mechanoregulation of endothelial cell function in thixotropic hydrogels *via* L-type Ca2+ channel and focal adhesion molecules for accelerated vascularization. Biomaterials 325, 123556. 10.1016/j.biomaterials.2025.123556 40690837

[B74] Liang C. Gao Y. Li J. Liang Q. Li M. Yang J. (2026). Bioengineered human pre-vascularized microtissues reconstruct vascular niche and regenerative microenvironment to enhance functional skeletal muscle regeneration. Biomaterials 326, 123645. 10.1016/j.biomaterials.2025.123645 40857913

[B75] Liao F. George A. Sui X. Semenov S. N. (2025). Autonomous spatiotemporal regulation of reversible hydrogel actuators by chemical reaction networks. Adv. Mater. 38, e16185. 10.1002/adma.202516185 41251508 PMC12862747

[B76] Liu C. Lou Y. Sun Z. Ma H. Sun M. Li S. (2023). 4D printing of personalized‐tunable biomimetic periosteum with anisotropic microstructure for accelerated vascularization and bone healing. Adv. Healthc. Mater. 12, 2202868. 10.1002/adhm.202202868 37171209

[B77] Liu W. Yang X. Wu S. Yu H. Sun M. Yu M. (2025). Shear-electrostatic synergistic hydrogels with biomimetic microchannels for vascularized maxillofacial bone regeneration. ACS Appl. Mat. Interfaces 17, 63057–63068. 10.1021/acsami.5c15956 41186524

[B78] Llamosí A. Szymański M. P. Szumna A. (2024). Molecular vessels from preorganised natural building blocks. Chem. Soc. Rev. 53, 4434–4462. 10.1039/D3CS00801K 38497833

[B79] Lor L. Tella E. Kottler D. Mahé E. Senet P. Monfort J. (2025). Leg ulcers associated with tyrosine kinase inhibitors of vascular endothelial growth factor receptors. Acad. Dermatol Venereol. Jdv 40, 70200. 10.1111/jdv.70200 41246915

[B80] Lu B. Ye M. Xia J. Zhang Z. Xiong Z. Zhang T. (2023). Electrical stimulation promotes the vascularization and functionalization of an engineered biomimetic human cardiac tissue. Adv. Healthc. Mater. 12, 2300607. 10.1002/adhm.202300607 36975154

[B81] Lu E. Yang X. Wang T. Huang X. Chen Y. Wang R. (2023). Biomimetic thermo-sensitive hydrogel encapsulating Hemangiomas stem cell derived extracellular vesicles promotes microcirculation reconstruction in diabetic wounds. Adv. Funct. Mater. 33, 2304250. 10.1002/adfm.202304250

[B82] Lu L. Wu Y. Lu C. Liang F. Gu S. Sun S. (2025). Molecular mechanism of Flt-1 protein and the regulation of monocytes modulate endothelial cell in wound healing sites *via* PGF/FLT1 signaling. Int. J. Biol. Macromol. 307, 142192. 10.1016/j.ijbiomac.2025.142192 40101823

[B83] Lu T. Xu Z. He Y. Shu M. Wang X. Zhang Y. (2025). 3D-Printed biomimetic ceramic scaffolds with photothermal/chemodynamic effects and ionic microenvironment for preventing tumor recurrence and promoting vascularized bone reconstruction. Adv. Funct. Mater. 35, 2418438. 10.1002/adfm.202418438

[B84] Ma W. Liu Z. Zhu T. Wang L. Du J. Wang K. (2024). Fabric-enhanced vascular graft with hierarchical structure for promoting the regeneration of vascular tissue. Adv. Healthc. Mater. 13, 2302676. 10.1002/adhm.202302676 38279911

[B85] Mendelson A. A. Ho E. Scott S. Vijay R. Hunter T. Milkovich S. (2022). Capillary module haemodynamics and mechanisms of blood flow regulation in skeletal muscle capillary networks: experimental and computational analysis. J. Physiol. 600, 1867–1888. 10.1113/JP282342 35067970

[B86] Mi B. Zhang J. Meng H. Xu Y. Xie J. Hao D. (2024). Laponite modified methacryloyl gelatin hydrogel with controlled release of vascular endothelial growth factor a for bone regeneration. Biochem. Biophys. Res. Commun. 733, 150714. 10.1016/j.bbrc.2024.150714 39326258

[B87] Mochizuki M. Güç E. Park A. J. Julier Z. Briquez P. S. Kuhn G. A. (2019). Growth factors with enhanced syndecan binding generate tonic signalling and promote tissue healing. Nat. Biomed. Eng. 4, 463–475. 10.1038/s41551-019-0469-1 31685999

[B88] Mu Y. Wang X. Du X. He P.-P. Guo W. (2024). DNA cryogels with anisotropic mechanical and responsive properties for specific cell capture and release. J. Am. Chem. Soc. 146, 5998–6005. 10.1021/jacs.3c12846 38379163

[B89] Ng A. C. T. Strudwick M. Van Der Geest R. J. Ng A. C. C. Gillinder L. Goo S. Y. (2018). Impact of epicardial adipose tissue, left ventricular myocardial fat content, and interstitial fibrosis on myocardial contractile function. Circ. Cardiovasc. Imaging 11, e007372. 10.1161/CIRCIMAGING.117.007372 30354491

[B90] Nguyen T. D. Nguyen T.-Q. Vo V. T. Nguyen T.-H. (2025). Advances in three-dimensional printing of hydrogel formulations for vascularized tissue and organ regeneration. J. Biomaterials Sci. Polym. Ed. 36, 1423–1465. 10.1080/09205063.2024.2449294 39899080

[B91] Nishiguchi A. Ohta M. Palai D. Ito S. Mori K. Akagi R. (2025). *In situ* forming supramolecular nanofiber hydrogel as a biodegradable liquid embolic agent for postembolization tissue remodeling. Adv. Healthc. Mater. 14, 2403784. 10.1002/adhm.202403784 39511876

[B92] Noly P.-E. Dorfmüller P. Fadel E. Mercier O. (2019). Capillary density in right ventricular myocardium in congenital heart disease. J. Heart Lung Transplant. 38, 328–331. 10.1016/j.healun.2018.12.001 30581050

[B93] Ohayon C. Gross N. Capucha T. Carasso S. Shkedy Y. Rachmiel A. (2025). Efficiency and outcomes in microvascular anastomosis: a meta-analysis of mechanical versus manual techniques. J. Cranio-Maxillofacial Surg. 53, 1720–1730. 10.1016/j.jcms.2025.07.015 40713241

[B94] Ozawa K. Muller M. A. Varlamov O. Tavori H. Packwood W. Mueller P. A. (2020). Proteolysis of Von Willebrand factor influences inflammatory endothelial activation and vascular compliance in atherosclerosis. JACC Basic Transl. Sci. 5, 1017–1028. 10.1016/j.jacbts.2020.08.009 33145464 PMC7591934

[B95] O’Grady B. J. McCall A. S. Cullison S. Chavarria D. Schrag M. S. Lippmann E. S. (2026). Human brain vasculature-on-a-chip model constructed with microvessels isolated from cryopreserved postmortem human brain tissue. Adv. Healthc. Mater. 15, e04167. 10.1002/adhm.202504167 41527268 PMC13058772

[B96] Pan X. Liu S. Long M. Peng R. Hu L. Yu L. (2025). Biomimetic vascular scaffolds *via* hybrid 3D printing-phase separation for vascularized cardiac tissue with enhanced perfusion and maturation. Biomater. Sci. 13, 4803–4815. 10.1039/D5BM00734H 40690254

[B97] Paradiso A. Volpi M. Martinez D. C. Jaroszewicz J. Costantini M. Swieszkowski W. (2024). Engineering biomimetic microvascular capillary networks in hydrogel fibrous scaffolds via microfluidics-assisted Co-Axial wet-spinning. ACS Appl. Mat. Interfaces 16, 65927–65941. 10.1021/acsami.4c15221 PMC11622188 39566902

[B98] Peng X. Wang X. Cheng C. Zhou X. Gu Z. Li L. (2020). Bioinspired, artificial, small-diameter vascular grafts with selective and rapid endothelialization based on an amniotic membrane-derived hydrogel. ACS Biomater. Sci. Eng. 6, 1603–1613. 10.1021/acsbiomaterials.9b01493 33455393

[B99] Qi J. Yan Y. Cheng B. Deng L. Shao Z. Sun Z. (2018). Enzymatic formation of an injectable hydrogel from a glycopeptide as a biomimetic scaffold for vascularization. ACS Appl. Mat. Interfaces 10, 6180–6189. 10.1021/acsami.7b18535 29380599

[B100] Qi J. Zheng S. Zhao N. Li Y. Zhang G. Yin W. (2023). A 3D bioprinted hydrogel multilevel arc vascular channel combined with an isomaltol core sacrificial process. Mater. Today Commun. 36, 106492. 10.1016/j.mtcomm.2023.106492

[B101] Qu X. Wang Q. Gan D. Sun H. Ni Z. Dong X. (2025). Tissue-adaptable hydrogel for mechanically compliant bioelectronic interfaces. Nano Lett. 25, 4759–4766. 10.1021/acs.nanolett.4c06165 40080799

[B102] Richalet J.-P. Hermand E. Lhuissier F. J. (2024). Cardiovascular physiology and pathophysiology at high altitude. Nat. Rev. Cardiol. 21, 75–88. 10.1038/s41569-023-00924-9 37783743

[B103] Rodriguez M. Kluge J. A. Smoot D. Kluge M. A. Schmidt D. F. Paetsch C. R. (2020). Fabricating mechanically improved silk-based vascular grafts by solution control of the gel-spinning process. Biomaterials 230, 119567. 10.1016/j.biomaterials.2019.119567 31761485 PMC6942127

[B104] Rovas G. Bikia V. Stergiopulos N. (2023). Design and computational optimization of compliance-matching aortic grafts. Front. Bioeng. Biotechnol. 11, 1179174. 10.3389/fbioe.2023.1179174 37456727 PMC10341153

[B105] Shao Z. Yin T. Jiang J. He Y. Xiang T. Zhou S. (2023). Wound microenvironment self-adaptive hydrogel with efficient angiogenesis for promoting diabetic wound healing. Bioact. Mater. 20, 561–573. 10.1016/j.bioactmat.2022.06.018 35846841 PMC9254353

[B106] Shen H. Zhang C. Meng Y. Qiao Y. Ma Y. Chen J. (2024). Biomimetic hydrogel containing copper sulfide nanoparticles and deferoxamine for photothermal therapy of infected diabetic wounds. Adv. Healthc. Mater. 13, 2303000. 10.1002/adhm.202303000 38063809

[B107] Soliman B. G. Longoni A. Wang M. Li W. Bernal P. N. Cianciosi A. (2023). Programming delayed dissolution into sacrificial bioinks for dynamic temporal control of architecture within 3D-bioprinted constructs. Adv. Funct. Mater. 33, 2210521. 10.1002/adfm.202210521

[B108] Sun Y. Zhang X. Wu T. Zhang Z. Yang R. Liu W. (2023). YAP-suppressive nanodrug crosslinked self-immunoregulatory polysaccharide injectable hydrogel for attenuating cardiac fibrosis to treat myocardial infarction. Adv. Funct. Mater. 33, 2214468. 10.1002/adfm.202214468

[B109] Sun H. Xing K. Liu K. Liu Y. Li Y. Sun Y. (2025). Bubble casting strategy to construct multifurcated hydrogel microtubes with adjustable dimensions and endothelialization. Biofabrication 17, 035027. 10.1088/1758-5090/adebb2 40609557

[B110] Szafron J. M. Heng E. E. Boyd J. Humphrey J. D. Marsden A. L. (2024). Hemodynamics and wall mechanics of vascular graft failure. ATVB 44, 1065–1085. 10.1161/ATVBAHA.123.318239 PMC11043008 38572650

[B111] Szklanny A. A. Machour M. Redenski I. Chochola V. Goldfracht I. Kaplan B. (2021). 3D bioprinting of engineered tissue flaps with hierarchical vessel networks (VesselNet) for direct host-to-implant perfusion. Adv. Mater. 33, 2102661. 10.1002/adma.202102661 34510579 PMC11468543

[B112] Tan M. Liu Y. Wang Y. Li Y. Wu C. Jiang Z. (2025). Wireless thermoelectric hydrogel recreates biomimetic electric field and angiogenic signal accelerating diabetic ulcer repair. Adv. Funct. Mater. 35, 2425610. 10.1002/adfm.202425610

[B113] Tang J. Su Z. Liu H. Xu C. Wang G. Dong X. (2025). The bionic code of small-diameter vascular grafts: Gelatin-sodium alginate hydrogel with dual network recapitulates vascular intima functions. Int. J. Biol. Macromol. 320, 146050. 10.1016/j.ijbiomac.2025.146050 40675307

[B114] Teng L. Song Y. Hu Y. Lu J. Dong C.-M. (2024). Biomimetic and wound microenvironment-modulating PEGylated glycopolypeptide hydrogels for arterial massive hemorrhage and wound prohealing. Biomacromolecules 25, 4317–4328. 10.1021/acs.biomac.4c00389 38829675

[B115] Tu C. Zhang Y. Xiao Y. Xing Y. Jiao Y. Geng X. (2022). Hydrogel-complexed small-diameter vascular graft loaded with tissue-specific vascular extracellular matrix components used for tissue engineering. Biomater. Adv. 142, 213138. 10.1016/j.bioadv.2022.213138 36219919

[B116] Wang X.-Y. Jin Z.-H. Gan B.-W. Lv S.-W. Xie M. Huang W.-H. (2014). Engineering interconnected 3D vascular networks in hydrogels using molded sodium alginate lattice as the sacrificial template. Lab. Chip 14, 2709–2716. 10.1039/C4LC00069B 24887141

[B117] Wang Z. Mithieux S. M. Weiss A. S. (2019). Fabrication techniques for vascular and vascularized tissue engineering. Adv. Healthc. Mater. 8, 1900742. 10.1002/adhm.201900742 31402593

[B118] Wang L. Chen Z. Yan Y. He C. Li X. (2021). Fabrication of injectable hydrogels from silk fibroin and angiogenic peptides for vascular growth and tissue regeneration. Chem. Eng. J. 418, 129308. 10.1016/j.cej.2021.129308

[B119] Wang X. Chan V. Corridon P. R. (2022). Acellular tissue-engineered vascular grafts from polymers: methods, achievements, characterization, and challenges. Polymers 14, 4825. 10.3390/polym14224825 36432950 PMC9695055

[B120] Wang Y. Kankala R. K. Ou C. Chen A. Yang Z. (2022). Advances in hydrogel-based vascularized tissues for tissue repair and drug screening. Bioact. Mater. 9, 198–220. 10.1016/j.bioactmat.2021.07.005 34820566 PMC8586021

[B121] Wang B. Wang X. Kenneth A. Drena A. Pacheco A. Kalvin L. (2023). Developing small-diameter vascular grafts with human amniotic membrane: long-term evaluation of transplantation outcomes in a small animal model. Biofabrication 15, 025004. 10.1088/1758-5090/acb1da 36626826

[B122] Wang Y. Xie Y. Xie X. Wu D. Wu H. Luo X. (2023). Compliant and robust tissue-like hydrogels via ferric ion-induced of hierarchical structure. Adv. Funct. Mater. 33, 2210224. 10.1002/adfm.202210224

[B123] Wang B. Liu J. Guo C. Bao X. Qi L. Yin J. (2024). Biomimetic dually cross-linked injectable poly(l-glutamic acid) based nanofiber composite hydrogels with self-healing, osteogenic and angiogenic properties for bone regeneration. Compos. Part B Eng. 280, 111492. 10.1016/j.compositesb.2024.111492

[B124] Wang T. Li X. Liu S. Zhang J. (2024). Stepwise co-assembled biomimetic hydrogel with antibacterial, angiogenesis, and tissue regeneration acceleration for diabetic wound healing. Mater. and Des. 244, 113149. 10.1016/j.matdes.2024.113149

[B125] Wang J. Wang Y. Lu M. Cao X. Xia M. Zhao M. (2025). Biomimetic triplet messenger RNA formulation in MXene hydrogel microneedles for wound healing. Aggregate 6, e700. 10.1002/agt2.700

[B126] Wang K. Lan X. Chen J. Wu Y. Zhu D. Kong X. (2025). One-step coaxial 3D printing of pre-vascularized skin organoid models with ADSC microspheres for enhanced wound healing. Adv. Sci. 13, e17409. 10.1002/advs.202517409 PMC12904007 41317393

[B127] Wang Q. Yang J. Meng J. Yu J. Li X. Ding B. (2025). Bioinspired hierarchical fiber-hydrogel dressing with dual immunomodulatory and angiogenic functions for diabetic wound healing. J. Control. Release 386, 114063. 10.1016/j.jconrel.2025.114063 40712890

[B128] Wang Y. Zhang Y. Zhou L. Li Y. Zu X. Zhang J. (2025). ADSC-derived extracellular vesicles loaded with VEGF mRNA delivered by antibacterial hydrogel promote angiogenesis and reconstruction of prefabricated flaps. Small 21, e08210. 10.1002/smll.202508210 41170665

[B129] Wei F.-L. Zhai Y. Wang T.-F. Zhao J.-W. Wang C.-L. Tang Z. (2024). Stem cell–homing biomimetic hydrogel promotes the repair of osteoporotic bone defects through osteogenic and angiogenic coupling. Sci. Adv. 10, eadq6700. 10.1126/sciadv.adq6700 39485837 PMC11529719

[B130] West-Livingston L. Lim J. W. Lee S. J. (2023). Translational tissue-engineered vascular grafts: from bench to bedside. Biomaterials 302, 122322. 10.1016/j.biomaterials.2023.122322 37713761

[B131] Wu X. Huo Y. Ci Z. Wang Y. Xu W. Bai B. (2022). Biomimetic porous hydrogel scaffolds enabled vascular ingrowth and osteogenic differentiation for vascularized tissue-engineered bone regeneration. Appl. Mater. Today 27, 101478. 10.1016/j.apmt.2022.101478

[B132] Wu M. Zhang Y. Zhao Y. Chu L. Meng X. Ye L. (2024). Photoactivated hydrogel therapeutic system with MXene-based nanoarchitectonics potentiates endogenous bone repair through reshaping the osteo-vascularization network. Small 20, 2403003. 10.1002/smll.202403003 39377343

[B133] Wu F. Chen H. Liu J. Pang Y. (2025). Generating structurally and functionally programmable hydrogels by biological membrane hybridization. Nat. Protoc. 21, 1597–1628. 10.1038/s41596-025-01247-4 40940524

[B134] Wu Q.-Y. Chen C.-Y. Li J.-C. Chen Z.-G. Chen S.-C. Xie C. (2026). Interpenetrated hierarchical polyzwitterionic hydrogels promote decellularized small-diameter vascular grafts regeneration. Chem. Eng. J. 529, 172764. 10.1016/j.cej.2026.172764

[B135] Xiao Z. Chen S. Zhang K. Jiang S. Qin H. Jian H. (2025). Biomimetic bilayer hydrogel loaded with SVF exosomes promotes scar-free healing of diabetic burn wounds. Chem. Eng. J. 520, 165672. 10.1016/j.cej.2025.165672

[B136] Xie R. Liang Z. Ai Y. Zheng W. Xiong J. Xu P. (2021). Composable microfluidic spinning platforms for facile production of biomimetic perfusable hydrogel microtubes. Nat. Protoc. 16, 937–964. 10.1038/s41596-020-00442-9 33318693

[B137] Xing M. Yu C. Wu Y. Wang L. Guan G. (2020). Preparation and characterization of a polyvinyl alcohol/polyacrylamide hydrogel vascular graft reinforced with a braided fiber stent. Text. Res. J. 90, 1537–1548. 10.1177/0040517519896753

[B138] Xu H. Su Y. Liao Z. Liu Z. Huang X. Zhao L. (2022). Coaxial bioprinting vascular constructs: a review. Eur. Polym. J. 179, 111549. 10.1016/j.eurpolymj.2022.111549

[B139] Xu Y. Xu C. He L. Zhou J. Chen T. Ouyang L. (2022). Stratified-structural hydrogel incorporated with magnesium-ion-modified black phosphorus nanosheets for promoting neuro-vascularized bone regeneration. Bioact. Mater. 16, 271–284. 10.1016/j.bioactmat.2022.02.024 35386320 PMC8965728

[B140] Yang P. Xie F. Zhu L. Selvaraj J. N. Zhang D. Cai J. (2024). Fabrication of chitin-fibrin hydrogels to construct the 3D artificial extracellular matrix scaffold for vascular regeneration and cardiac tissue engineering. J. Biomed. Mater. Res. 112, 2257–2272. 10.1002/jbm.a.37774 39007419

[B141] Yang R. Tu Z. Chen X. Wu X. (2024). Highly stretchable, robust, sensitive and wearable strain sensors based on mesh-structured conductive hydrogels. Chem. Eng. J. 480, 148228. 10.1016/j.cej.2023.148228

[B142] Yang Y. Wang J. Huang S. Li M. Chen J. Pei D. (2024). Bacteria-responsive programmed self-activating antibacterial hydrogel to remodel regeneration microenvironment for infected wound healing. Natl. Sci. Rev. 11, nwae044. 10.1093/nsr/nwae044 38440214 PMC10911815

[B143] Yao Y. Pohan G. Cutiongco M. F. A. Jeong Y. Kunihiro J. Zaw A. M. (2023). *In vivo* evaluation of compliance mismatch on intimal hyperplasia formation in small diameter vascular grafts. Biomater. Sci. 11, 3297–3307. 10.1039/D3BM00167A 36943136 PMC10160004

[B144] Ye T. Chai M. Wang Z. Shao T. Liu J. Shi X. (2024). 3D-Printed hydrogels with engineered nanocrystalline domains as functional vascular constructs. ACS Nano 18, 25765–25777. 10.1021/acsnano.4c08359 39231281

[B145] Yrigoin K. Davis G. E. (2024). Selective mural cell recruitment of pericytes to networks of assembling endothelial cell-lined tubes. Front. Cell Dev. Biol. 12, 1389607. 10.3389/fcell.2024.1389607 38961866 PMC11219904

[B146] Yu Y. Xiao H. Tang G. Wang H. Shen J. Sun Y. (2023). Biomimetic hydrogel derived from decellularized dermal matrix facilitates skin wounds healing. Mater. Today Bio 21, 100725. 10.1016/j.mtbio.2023.100725 37483381 PMC10359665

[B147] Yue T. Liu N. Liu Y. Peng Y. Xie S. Luo J. (2019). On-Chip construction of multilayered hydrogel microtubes for engineered vascular-like microstructures. Micromachines 10, 840. 10.3390/mi10120840 31805688 PMC6953073

[B148] Zeng Y. Wang C. Lei K. Xiao C. Jiang X. Zhang W. (2023). Multifunctional MOF-based microneedle patch with synergistic chemo-photodynamic antibacterial effect and sustained release of growth factor for chronic wound healing. Adv. Healthc. Mater. 12, 2300250. 10.1002/adhm.202300250 36940484

[B149] Zhang W. Liu Y. Kassab G. S. (2007). Viscoelasticity reduces the dynamic stresses and strains in the vessel wall: implications for vessel fatigue. Am. J. Physiol.-Heart Circulatory Physiol. 293, H2355–H2360. 10.1152/ajpheart.00423.2007 17604330

[B150] Zhang Y. Li X. S. Guex A. G. Liu S. S. Müller E. Malini R. I. (2017). A compliant and biomimetic three-layered vascular graft for small blood vessels. Biofabrication 9, 025010. 10.1088/1758-5090/aa6bae 28382923

[B151] Zhang L. Xiang Y. Zhang H. Cheng L. Mao X. An N. (2020). A biomimetic 3D-self-forming approach for microvascular scaffolds. Adv. Sci. 7, 1903553. 10.1002/advs.201903553 32382485 PMC7201264

[B152] Zhang C. Xie Q. Cha R. Ding L. Jia L. Mou L. (2021). Anticoagulant hydrogel tubes with poly(ɛ-caprolactone) sheaths for small-diameter vascular grafts. Adv. Healthc. Mater. 10, 2100839. 10.1002/adhm.202100839 34218526

[B153] Zhang M. Wang G. Zhang X. Zheng Y. Lee S. Wang D. (2021). Polyvinyl alcohol/chitosan and polyvinyl Alcohol/Ag@MOF bilayer hydrogel for tissue engineering applications. Polymers 13, 3151. 10.3390/polym13183151 34578053 PMC8468989

[B154] Zhao N. Pessell A. F. Zhu N. Searson P. C. (2024). Tissue-engineered microvessels: a review of current engineering strategies and applications. Adv. Healthc. Mater. 13, 2303419. 10.1002/adhm.202303419 PMC11338730 38686434

[B155] Zheng M.-H. Shan S.-K. Lin X. Xu F. Wu F. Guo B. (2023). Vascular wall microenvironment: exosomes secreted by adventitial fibroblasts induced vascular calcification. J. Nanobiotechnol. 21, 315. 10.1186/s12951-023-02000-3 PMC10478424 37667298

[B156] Zheng Z. Sun J. Wang J. He S. Liu Z. Xie J. (2024). Enhancing myocardial infarction treatment through bionic hydrogel-mediated spatial combination therapy via mtDNA-STING crosstalk modulation. J. Control. Release 371, 570–587. 10.1016/j.jconrel.2024.06.015 38852624

[B157] Zhong Q. Wang D. Mai H. Chen R. Xu Y. Lei M. (2024). Injectable thermo-responsive poloxamer hydrogel/methacrylate gelatin microgels stimulates bone regeneration through biomimetic programmed release of SDF-1a and IGF-1. Int. J. Biol. Macromol. 271, 132742. 10.1016/j.ijbiomac.2024.132742 38821297

[B158] Zhou L. Li Y. Tu Q. Wang J. (2023). A 3D printing mold method for rapid fabrication of artificial blood vessels. Colloids Surfaces A Physicochem. Eng. Aspects 662, 130952. 10.1016/j.colsurfa.2023.130952

[B159] Zhou Z. Liu Y. Li W. Zhao Z. Xia X. Liu J. (2024). A self-adaptive biomimetic periosteum employing nitric oxide release for augmenting angiogenesis in bone defect regeneration. Adv. Healthc. Mater. 13, 2302153. 10.1002/adhm.202302153 37922941

[B160] Zhou E. Zhu Y. Chen G. Liu D. Zhao L. Wang M. (2025). Bioactive nanocomposite hydrogel with synergistic antibacterial and cytoprotective functions: multi-target keratinocyte modulation for infected wound regeneration. Chem. Eng. J. 520, 165748. 10.1016/j.cej.2025.165748

[B161] Zhou M. Wang Z. Li M. Chen Q. Zhang S. Wang J. (2025). Passivated hydrogel interface: armor against foreign body response and inflammation in small-diameter vascular grafts. Biomaterials 317, 123010. 10.1016/j.biomaterials.2024.123010 39724767

[B162] Zohar B. Debbi L. Machour M. Nachum N. Redenski I. Epshtein M. (2023). A micro-channel array in a tissue engineered vessel graft guides vascular morphogenesis for anastomosis with self-assembled vascular networks. Acta Biomater. 163, 182–193. 10.1016/j.actbio.2022.05.026 35597433

